# Comparative Phytochemistry of Polyacetylenes of the Genus *Artemisia* (Asteraceae): Compounds with High Biological Activities and Chemotaxonomic Significance

**DOI:** 10.3390/molecules30030537

**Published:** 2025-01-24

**Authors:** Harald Greger

**Affiliations:** Chemodiversity Research Group, Faculty of Life Sciences, University of Vienna, Rennweg 14, A-1030 Wien, Austria; harald.greger@univie.ac.at

**Keywords:** polyacetylenes, polyynes, spiroketal enol ethers, aromatic acetylenes, isocoumarins, structural relationships, chemotaxonomy, biological activities, antifungal, insecticidal, nematicidal, cytotoxic, inhibition of superoxide generation, allelopathy

## Abstract

In spite of the many chemical reports on polyacetylenes of the genus *Artemisia*, combined conclusions regarding their distribution and biological functions are widely missing. The aim of the present review was to arrange the diversity of polyacetylenes in the genus following biogenetic aspects and group them together into characteristic structural types. The co-occurrence of the dehydrofalcarinol type with the aromatic capillen-isocoumarin type represents a characteristic biogenetic trend, clearly segregating species of the subgenus Dracunculus from those of the subgenera *Artemisia* and Absinthium, distinguished by the spiroketal enol ether and/or linear triyne type. Various accumulation trends toward specific structures additionally contribute to a more natural species grouping within the subgenera. Biological activities were reported for all four structural types, ranging from antifungal, insecticidal, nematicidal, and cytotoxic properties to allelopathic effects. Of particular interest were their remarkable cytotoxic potencies, from which the very high values of dehydrofalcarin-3,8-diol may be associated with the pronounced affinity of this type to form extremely stable bonds to proteins acting in signaling pathways. The aromatic acetylene capillin inhibited the viability of various tumor cells in a dose- and time-dependent manner. Its potent apoptosis-inducing activity was induced via the mitochondrial pathway. A group of spiroketal enol ethers was identified as inhibitors of PMA-induced superoxide generation. Among them, the epoxide of the isovalerate ester exhibited the highest potency. The ecological impact of acetylene formation was made apparent by the allelopathic effects of DME of the linear triyne type, and the aromatic capillen by inhibiting seed germination and growth of widespread weeds.

## 1. Introduction

### 1.1. Biosynthetic Aspects

Naturally occurring polyacetylenes (=polyynes) are characterized by the formation of triple bonds that can be derived from different metabolic routes. As derivatives of the isoprenoid, polyketide, or fatty acid pathway, they are widely distributed in different organisms, including bacteria, marine invertebrates, fungi, plants, and animals. However, the majority of polyacetylenes were shown to be derived from fatty acids and were mostly isolated from the three related plant families Asteraceae, Apiaceae, and Araliaceae [1,2,3,4,5,6]. The highest number with more than 1100 compounds was reported for the Asteraceae, where the three tribes Anthemideae, Heliantheae, and Cynareae are especially rich in structural variation. With a number of well-known medicinal plants, such as species of the genera *Artemisia*, *Achillea*, *Matricaria*, and *Tanacetum*, Anthemideae represents the best-investigated tribe showing the highest diversity of polyacetylenes. Here, of special interest is the ability to synthesize aromatic rings from linear acetylenic precursors and the formation of unique spiroketal enol ethers [7,8]. All these tribus-specific structures were found in the large genus *Artemisia* comprising around 500 species. Together with other chemical features, such as different lengths of carbon chains and the number of conjugated triple bonds, they provide important chemotaxonomic criteria for an infrageneric grouping [1,9]. Due to the typical UV spectra of polyacetylenes, broad-based UV-HPLC comparisons of lipophilic extracts of *Artemisia* species provided information about the accumulation and distribution of characteristic derivatives [10,11,12,13].

**Figure 1:**
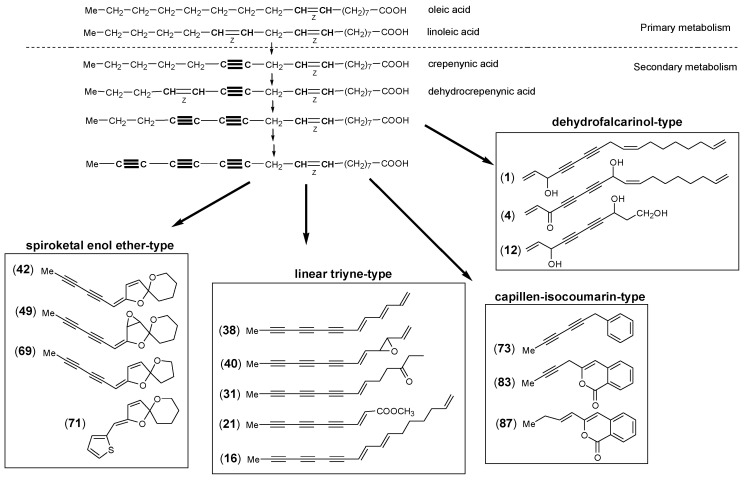
Desaturation of fatty acids and transformation to four structural types of polyacetylenes in the genus *Artemisia*.

Based on extensive feeding experiments with ^14^*C*- and ^3^*H*-labeled precursors, the main line of biosynthetic sequence of the fatty-acid-derived acetylenes has already been determined. Starting with C_18_-crepenynic acid, as the first monoacetylenic precursor in the Asteraceae, increasing desaturation at the distal part (near the methyl end) of the molecule leads to a group of methyl-triynes typical for many *Artemisia* species. Structural diversification is further created by oxidative chain-shortening and cyclization processes at the proximal part (near the carboxy group) [1,4,14,15]. A significant diversion step in the main biosynthetic route is the formation of *C*_17_-diynes of the dehydrofalcarinol type (Figure 1). This biogenetic trend can be regarded as a conservative chemical feature also known from other genera of the Anthemideae and even other tribes of the family Asteraceae. Moreover, with structurally corresponding, but more saturated, falcarinol (=panaxynol)-type derivatives [16], this group of *C*_17_-acetylenes was shown to represent also a significant chemical character of the two families Araliaceae and Apiaceae [17,18,19,20].

### 1.2. Bioactive Properties

Starting with reports on the toxic acetylene cicutoxin, a *C*_17_-compound from *Cicuta virosa* L. (Apiaceae) [21], and the antifungal activity of the aromatic acetylene capillin (**77**) from *Artemisia capillaris* Thunb. [22], followed by the discovery of the fish poison ichthyothereol (**35**), a *C*_14_-acetylenic tetrahydropyrane from *Ichthyothere terminalis* (Spreng.) Malme (Asteraceae) [23], different types of polyacetylenes were shown to possess a wide range of biological activities [24]. Apart from aromatic derivatives [25,26,27,28] and spiroketal enol ethers [29,30,31,32,33,34,35,36], especially *C*_17_-acetylenes of the falcarinol type exhibited pronounced antifungal [37,38,39,40,41,42,43,44,45,46], insecticidal [42,43,45,46,47,48], nematicidal [46,49,50,51], antibacterial [52,53,54], and cytotoxic effects [20,52,55,56,57,58,59,60,61,62,63]. The ecological impact of acetylene formation was made apparent by their function as phytoalexins [40,64,65] and by their photosensitizing [26,47,66,67,68] and allelopathic effects [69,70,71]. Improved analytical methods and the progress in evaluating bioassays greatly stimulated further investigations in this class of compounds, as summarized in a series of reviews [4,6,18,24,59,72,73,74,75].

### 1.3. Phytochemical Characters

Species of the genus *Artemisia* are among the most widespread and widely used medicinal plants in the world. Consequently, they have been the subject of many phytochemical studies focusing mainly on monoterpenes, sesquiterpene lactones, flavonoids, lignans, coumarins, and polyacetylenes [76,77,78,79,80,81,82,83,84]. The first naturally occurring polyacetylene, a methyl *n*-decen-triyonate or dehydromatricaria ester (DME) (**21**), was obtained by steam distillation from the roots of *Artemisia vulgaris* L. (“common mugwort”) almost 200 years ago [85]. Although different polyacetylenes were later also isolated by methods of essential oil production, careful lipophilic solvent extraction at room temperature was meanwhile preferred in view of the unstable derivatives [86]. Considering the biogenetic connections and different distribution within the genus, the polyacetylenes of *Artemisia* can be grouped into four structural types representing derivatives of dehydrofalcarinol, capillen-isocoumarin, spiroketal enol ether, and linear triyne (Figure 1). The aim of the present review is to provide an updated overview of the structural diversity of polyacetylenes in the genus *Artemisia* and to summarize the many publications describing the various bioactivities. In addition, different distributions of the structural types within the genus should demonstrate to what extent polyacetylenes can serve as chemotaxonomic markers.

## 2. Structural Types

The conversion of the *C*-12 double bond of linoleic acid into the triple bond of crepenynic acid represents the first step in the formation of polyacetylenes in the Asteraceae. Further desaturation steps, performed by desaturase and acetylenase activities [4], lead to compounds with up to three conjugated triple bonds, widely distributed in *Artemisia*. An important biosynthetic trend diverges at the diyne level leading to the dehydrofalcarinol type, mainly occurring as *C*_17_-derivatives. Chain-shortening of the *C*_18_-triyne fatty acids, involving β- and α-oxidation, results in the formation of *C*_14_- and *C*_13_- intermediates, respectively, which are further modified either to the widespread linear triynes or to the deviating structures of the spiroketal enol ether and the capillen-isocoumarin type (Figure 1). Besides this major desaturation pathway, additional side reactions are proposed for the formation of the terminal vinyl end both in the linear triynes and in the dehydrofalcarinol group.

### 2.1. Dehydrofalcarinol Type

**Scheme 1:**
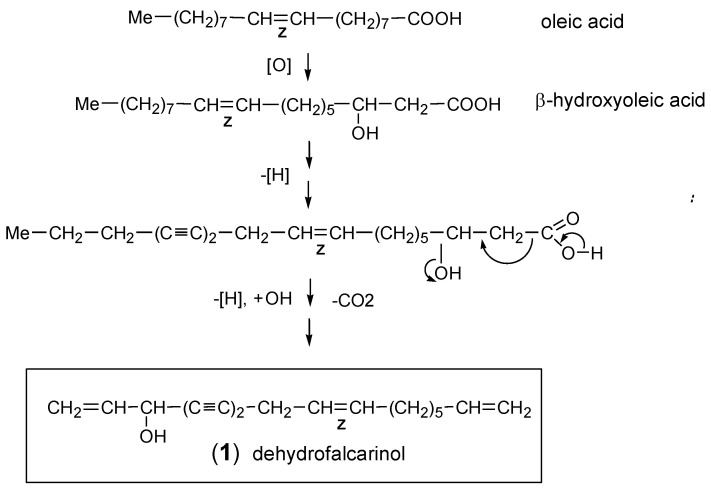
Biosynthesis of dehydrofalcarinol.

**Figure 2:**
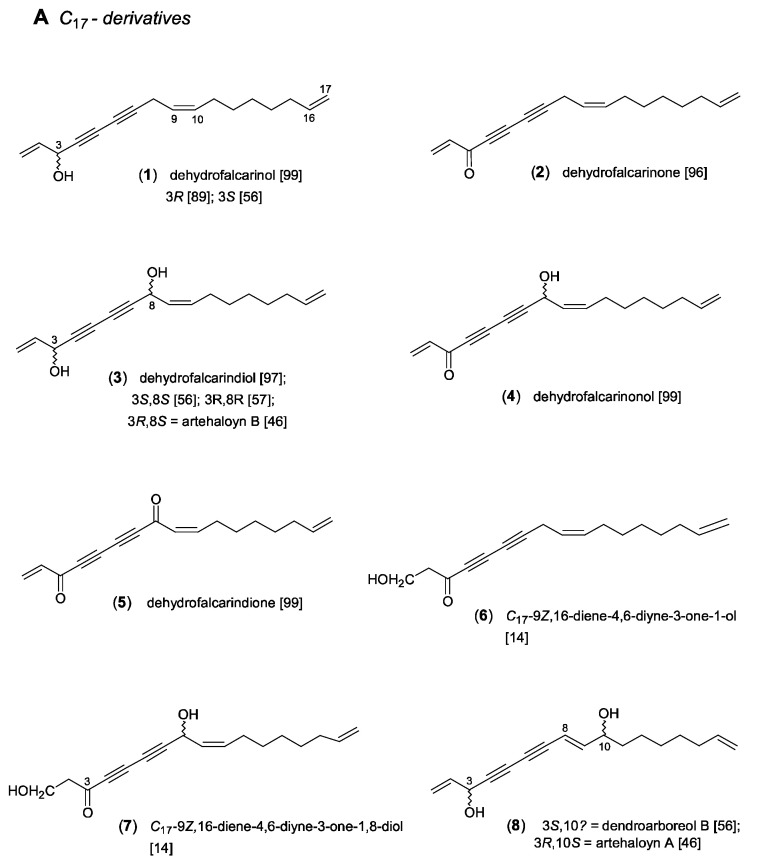

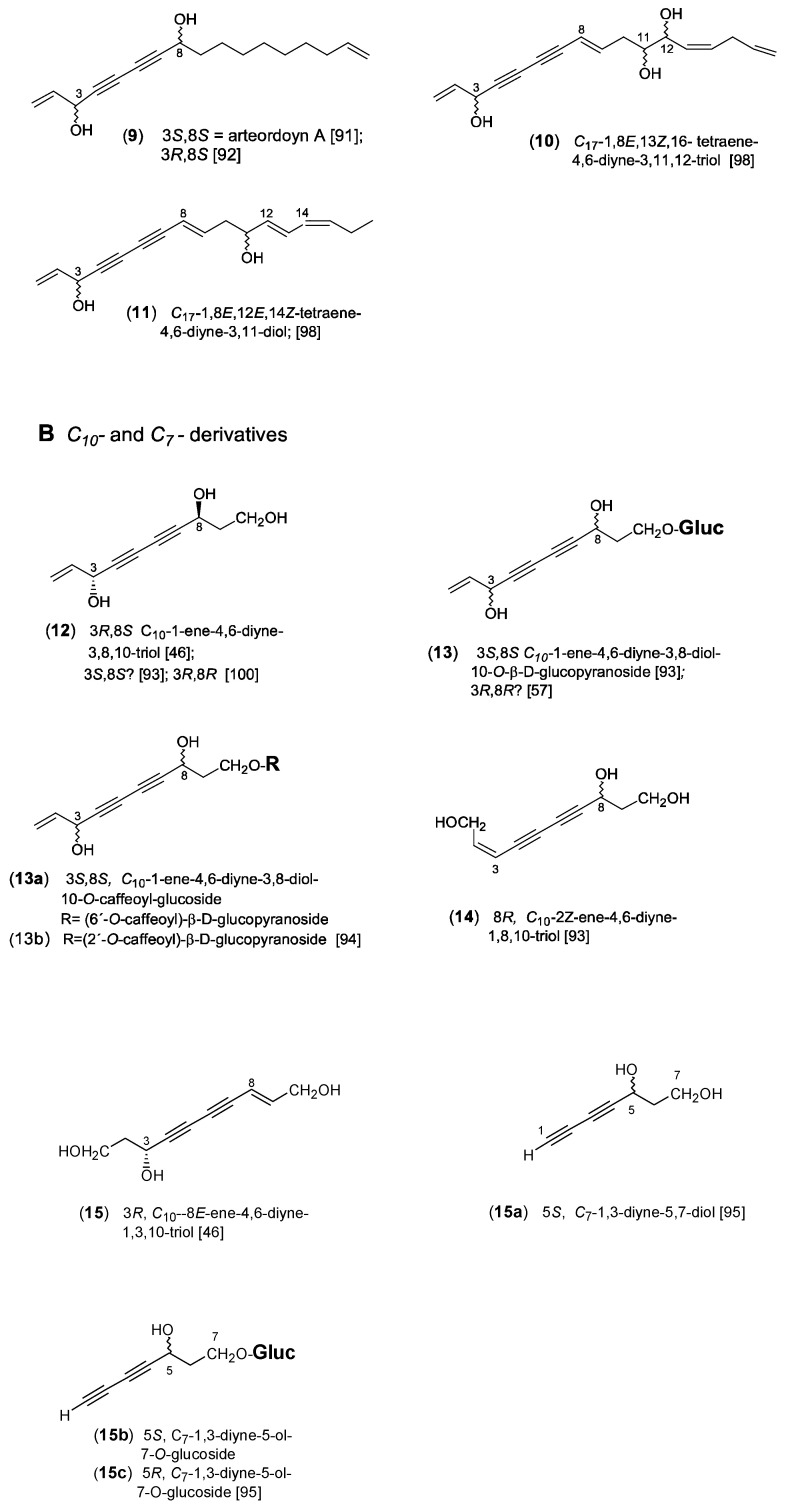
Dehydrofalcarinol-type acetylenes [14,46,56,57,89,91,92,93,94,95,96,97,98,99,100]. (**A**) *C*_17_-derivatives; (**B**) *C*_10_- and *C*_7_-derivatives.

The polyacetylenes of the dehydrofalcarinol type (Figure 2) are generally characterized by a *C*_17_-1,9,16-triene-4,6-diyne basic structure linked with oxygen substituents at the *C*-3 and *C*-8 positions. As demonstrated in Scheme 1, β-hydroxyoleic acid was proposed as a biosynthetic precursor. After desaturation and expulsion of *CO*_2_, dehydration leads to the characteristic vinyl end group [87]. The formation of the second double bond at the other end of the carbon chain and the adjacent oxygen substitution can be explained by desaturation and hydroxylation [4]. As shown in Figure 2, the central (*Z*)-configurated double bond at *C*-9, derived from oleic acid, divides the molecule into a polar and an apolar region. The former is highly unsaturated and characterized by oxygen functionalities, while the latter consists of a saturated carbon chain terminated by a vinyl group. This double bond at *C*-16 of the dehydrofalcarinol derivatives is of particular chemotaxonomic significance, representing a typical chemical feature of the family Asteraceae. By contrast, in the corresponding structures of falcarinol (=panaxynol) derivatives [16] of the related families Araliaceae, Apiaceae, and Pittosporaceae [88], the apolar region is characterized by a fully saturated carbon chain. Apart from varying oxo or hydroxy groups in the *C*-3 and *C*-8 positions, different stereochemistries of the *OH* groups were also reported. However, the assignment of the absolute configurations appeared problematic. Whilst for dehydrofalcarinol (**1**), isolated from *A. dracunculus* L., the 3*R* configuration was determined [89], different enantiomers are known from dehydrofalcarindiol (**3**). In accordance with falcarindiol [90], a 3*R*,8*S* configuration was also assigned for (**3**) in *A. halodendron* Turcz. ex Bess., named artehaloyn B [46], whereas 3*R*,8*R* was determined for (**3**) in *A. monosperma* Del. [57]. In addition, dehydrofalcarindiol (**3**) was also shown to occur as a 3*S*,8*S*-configurated isomer in *Dendropanax arboreus* of the family Araliaceae [56]. Two stereoisomers were also reported for the *C*_17_-1,16-diene-4,6-diyne-3,8-diol (**9**), from which the 3*S*,8*S*-isomer, named arteordoyn A, was isolated from *A. ordosica* Krasch. [91], and the 3*R*,8*S*-isomer from *A. halodendron* [92].

Further structural modifications, shown in compounds (**8**–**11**) (Figure 2), can be explained by intra-chain oxidation and conjugase activity [4]. Chain-shortening to the structurally related *C*_10_-derivatives (**12**–**15**) (Figure 2) is supposed to be formed either via β-oxidation steps, or, regarding the co-existence of compounds (**8**) and (**15**) in *A. halodendron* [46], by a mid-chain cleavage [1]. Since only few glycosylated acetylenes are known so far, the formation of 10-*O*-glucoside (**13**) in the aerial parts of the three related species *A. monosperma* [57], *A. capillaris* [93], and *A. scoparia* [94] is of chemotaxonomic interest. In *A. scoparia*, two isomeric caffeoylated glucosides (**13a**,**b**) additionally contribute to the structural diversity of this type of acetylenes [94]. Moreover, from the aerial parts of *A. dracunculus*, three *C*_7_-diynes (**15a**–**c**) were isolated [95]. With respect to the already known tri-hydroxylated *C*_10_-diyne (**15**), they may be regarded as a result of an additional C_3_-cleavage.

### 2.2. Linear Triyne Type

#### 2.2.1. Centaur X_3_

**Figure 3:**
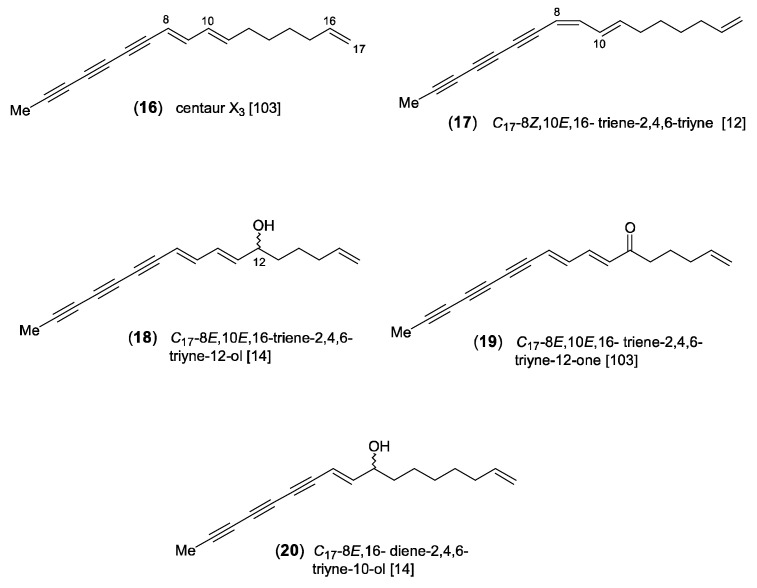
Centaur X_3_ and related *C*_17_-derivatives [12,14,103].

The widespread *C*_17_-acetylene centaur X_3_ (**16**) was first discovered in aerial parts of *Centaurea cyanus* L. (“cornflower”) by Löfgren [101] and was later also found in the essential oil of the roots of *A. vulgaris* [85]. The structure was elucidated by Bohlmann et al. [102] and is characterized by a methyl triyne-diene group combined with an isolated terminal double bond (Figure 3). Its biosynthesis was proposed to involve β-hydroxyoleic acid as precursor [1]. The dominating (8*E*,10*E*)-isomer (**16**) was shown to co-occur with a second mono-(*Z*)-isomer which was initially presumed to be (8*E*,10*Z*)-configurated. However, in a reinvestigation of *A. vulgaris* roots, a close inspection of the ^1^H-NMR spectra showed that the second isomer (**17**) is distinguished by (8*Z*,10*E*) configuration. The double bond between *C*-8 and *C*-9 near the center of the molecule and in conjugation to three conjugated triple bonds was assumed to (photo-)isomerize easily. Consequently, it is difficult to arrive at a decision concerning the original distribution of corresponding (*E*)- or (*Z*)-isomers in living plants [12]. The compounds (**18**) and (**19**) represent oxidation products of centaur X_3_ (**16**), while the alcohol (**20**), isolated from *A. selengensis* Turcz. [103], can be regarded as a biogenetic precursor (Figure 3).

#### 2.2.2. Dehydromatricaria Ester (DME)

**Scheme 2:**
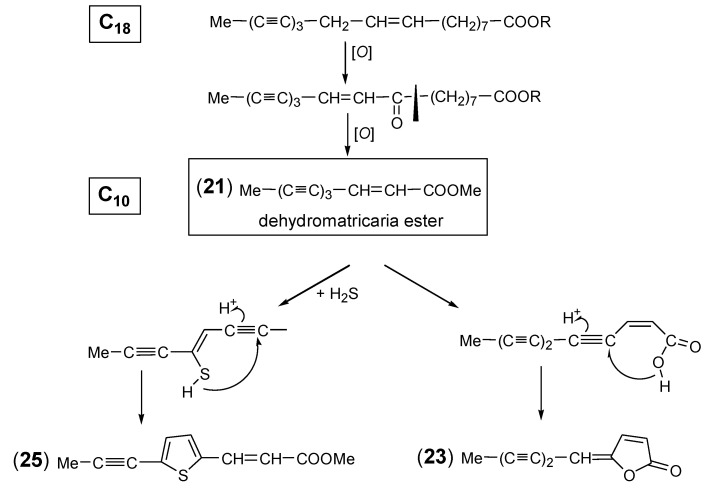
Biosynthesis of dehydromatricaria ester (DME).

**Figure 4:**
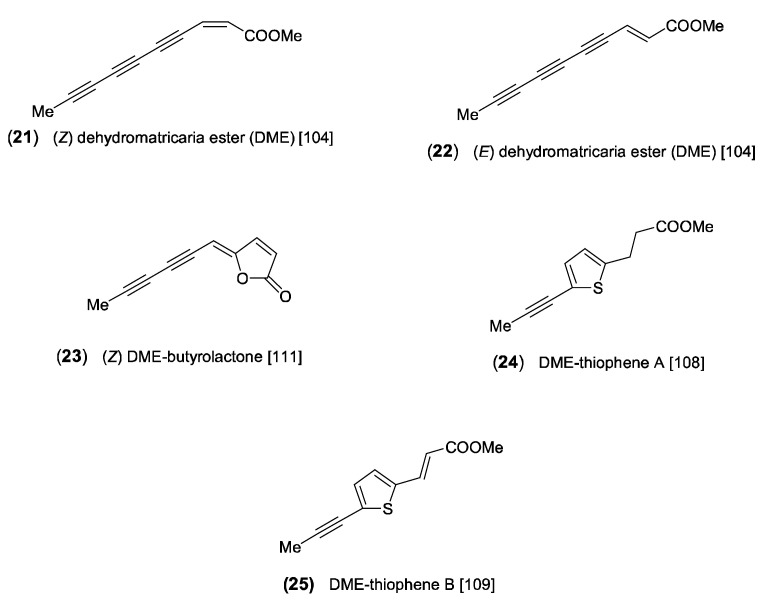
*C*_10_-acetylenes derived from dehydromatricaria ester (DME) [104,108,109,111].

A frequently occurring polyacetylene of *Artemisia* is the methyl ester of *C*_10_-triyne-enoic acid, named dehydromatricaria ester (DME). The (*Z*)-isomer (**21**) was discovered as a readily crystallizing compound in the essential oil of the roots of *A. vulgaris* [85], and its structure was later determined by Bohlmann and Mannhardt [104] (Figure 4). The formation of DME was proposed to be the result of a direct C_8_-cleavage from a C_18_-triynoic acid precursor (Scheme 2). The (*E*)-isomer (**22**), initially obtained by synthesis, was originally isolated from the genus *Tripleurospermum* (published as *Matricaria*) [105] and was later found to be very common in the tribe Anthemideae [1]. Examining the seasonal variation of (*Z*/*E*)-isomerism, it was shown that the (*Z*)-isomer (**21**) is the dominating compound in the roots of *A. vulgaris*, with increasing amounts from July to October [106]. However, a comparative analysis of 78 different samples of the *Artemisia* “Vulgares” group revealed a species-specific trend of accumulating either the (*Z*) (**21**)- or the (*E*)-isomer (**22**), suggesting a genetically fixed mechanism [13]. The isomers of a related butyrolactone were reported to be also common in the tribe Anthemideae [1]. However, in *Artemisia*, only small amounts of the (*Z*)-isomer (**23**) [107] (Figure 4) could be detected so far in the “Vulgares” group [13]. Further structural variation of DME is created by the incorporation of sulfur, leading to the formation of thiophenes [108,109] (Scheme 2). The dihydro-derivative DME-thiophene A (**24**) was isolated from the roots of two European samples of *A. absinthium* L. (“wormwood”) [108]. It was later also detected in a sample originating from China together with DME-thiophene B (**25**). In this case, the two DME derivatives were reported to coexist with a series of *C*_12_-dithiophenes [109]. However, this type of thiophene is unknown from the genus *Artemisia* and has not been detected in the tribe Anthemideae. Moreover, regarding the occurrence of DME-derived thiophenes in *A. absinthium*, the biogenetically deviating structure of a new *C*_10_-acetylenic thiophene in this species [110] should be reconsidered.

#### 2.2.3. “*Artemisia* Ketone” (AK) and Related C_14_-Derivatives

**Scheme 3:**
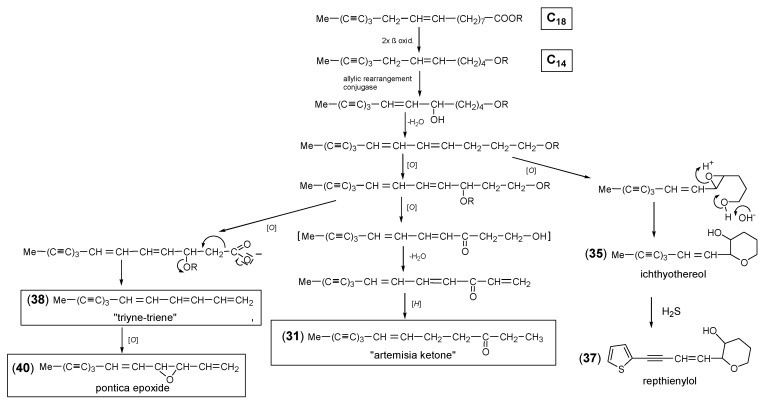
Proposed formation of “*Artemisia* ketone” (AK) (**31**).

**Figure 5:**
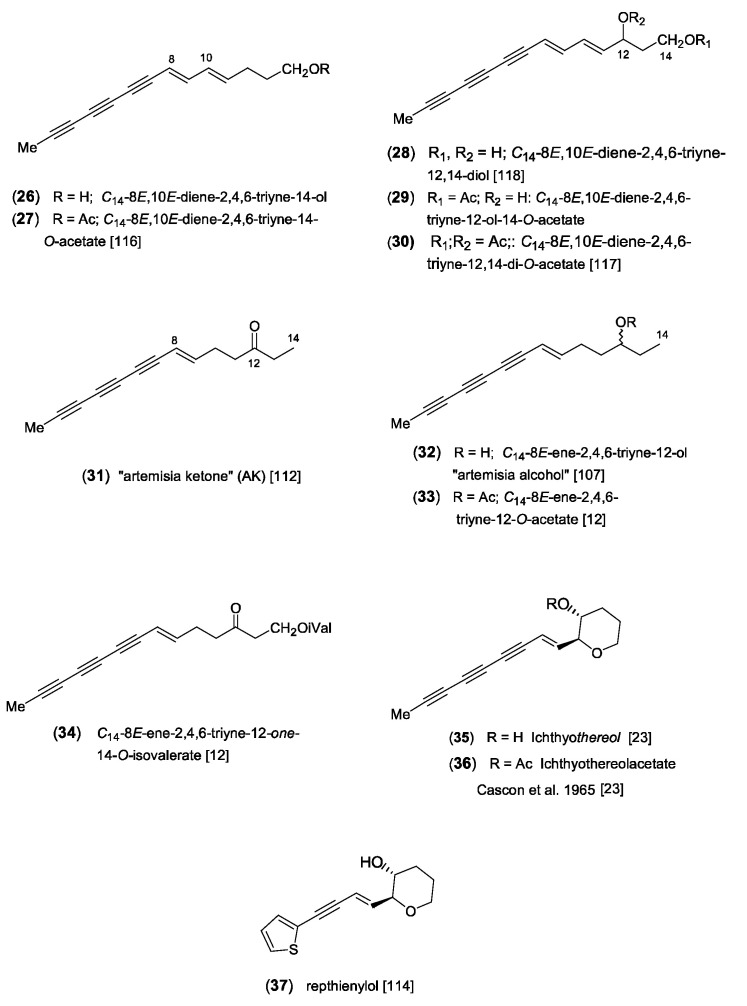
“*Artemisia* ketone” (AK) and related *C*_14_-derivatives [12,23,107,112,114,116,117,118].

**Figure 6:**
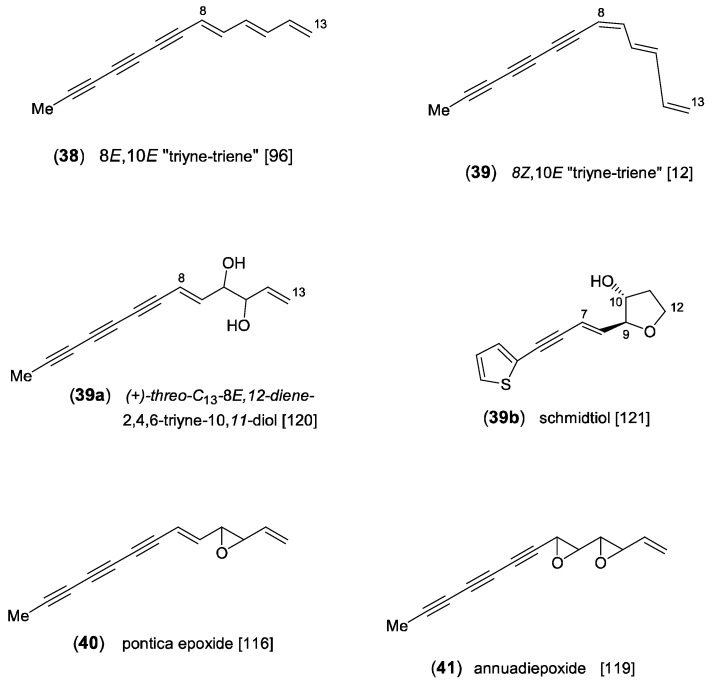
“Triyne-triene”, pontica epoxide, and related derivatives [12,96,116,119,120,121].

The *C*_14_-triyne-ene ketone (**31**) represents a typical polyacetylene of the tribe Anthemideae also widespread in the genus *Artemisia* [1,12,13]. Together with centaur X_3_ (**16**) and DME (**21**), it was originally detected in the roots of *A. vulgaris*, and its UV spectrum suggested the presence of a triyne-ene chromophore [85]. Due to the keto group at an unusual position, the structure could be elucidated only after extensive experiments and was named “artemisia ketone” (AK) [112]. However, this trivial name was already used first for an irregular monoterpene. The biosynthetic sequence leading to an oxygen in a not-allylic position was proposed by Bohlmann et al. [1,113] (Scheme 3). The corresponding alcohol (**32**) was first described for the roots of *Anacyclus pyrethrum* [107] and was also found to be common in the tribe Anthemideae. Its acetate (**33**) and the isovalerate ester of the structurally related keto-alcohol (**34**) were isolated from different species of the *Artemisia* “Vulgares” group [12,13]. Important key intermediates in this biosynthetic pathway are the *C*_14_-triyne-diene-alcohols (**26**) and (**28**) (Figure 5). They lead to the formation of the tetrahydropyrane ichthyothereol (**35**), its acetate (**36**) [23], and the thiophene repthienylol (**37**) from *A. reptans* C. Sm. [114] on the one hand and to the chemotaxonomically important *C*_13_-derivatives “triyne-triene” (**38**) and pontica epoxide (**40**) on the other. The alcohol (**26**) and its acetate (**27**) are widely distributed in the tribe Anthemideae [7] and were frequently detected in the *Artemisia* “Vulgares” group [13]. The co-existence of the two acetates (**27**) and (**30**) in *A. afra* Jacq. together with compounds (**31**, **36**, **38**) and (**40**) (Figure 5 and Figure 6) [115] underlines the biogenetic connections demonstrated in Scheme 3.

#### 2.2.4. “Triyne-Triene” and Pontica Epoxide (PE)

The *C*_13_-acetylene “triyne-triene” (**38**) is characterized by a fully conjugated system derived from a triyne-diene-diol intermediate (Scheme 3). It was initially isolated from the roots of *Achillea ptarmica* L., and its characteristic UV spectrum was detected in many species of the tribe Anthemideae [96]. In the genus *Artemisia*, compound (**38**) was frequently reported for the “Vulgares” [13] and especially characterized species of the “Abrotana” group. As already pointed out for centaur X_3_ (**16**), the central (*E)*-configurated double bond, conjugated with three triple bonds, easily isomerizes to the (*Z*)-isomer (**39**) [12]. The widespread oxidation product (**40**) was detected in different genera of the Anthemideae and represents the first naturally occurring polyacetylene-epoxide. It was first isolated from the underground parts of *A. pontica* L. and named pontica epoxide (PE) [116]. From the aerial parts of *A. annua* L., pontica epoxide (**40**) was also isolated as a major component along with a highly unstable diepoxide, named annuadiepoxide (**41**) [119]. In addition, the aerial parts were shown to contain the corresponding diol (**39a**) [120]. The inclusion of sulfur and demethylation lead to the *C*_12_-thiophene schmidtiol (**39b**), isolated as a minor component from the aerial parts of *A. schmidtiana* Maxim. Biogenetically, it is probably derived from a corresponding 12-hydroxy-9,10-epoxide precursor [121] (Figure 6).

### 2.3. Spiroketal Enol Ether Type

**Figure 7:**
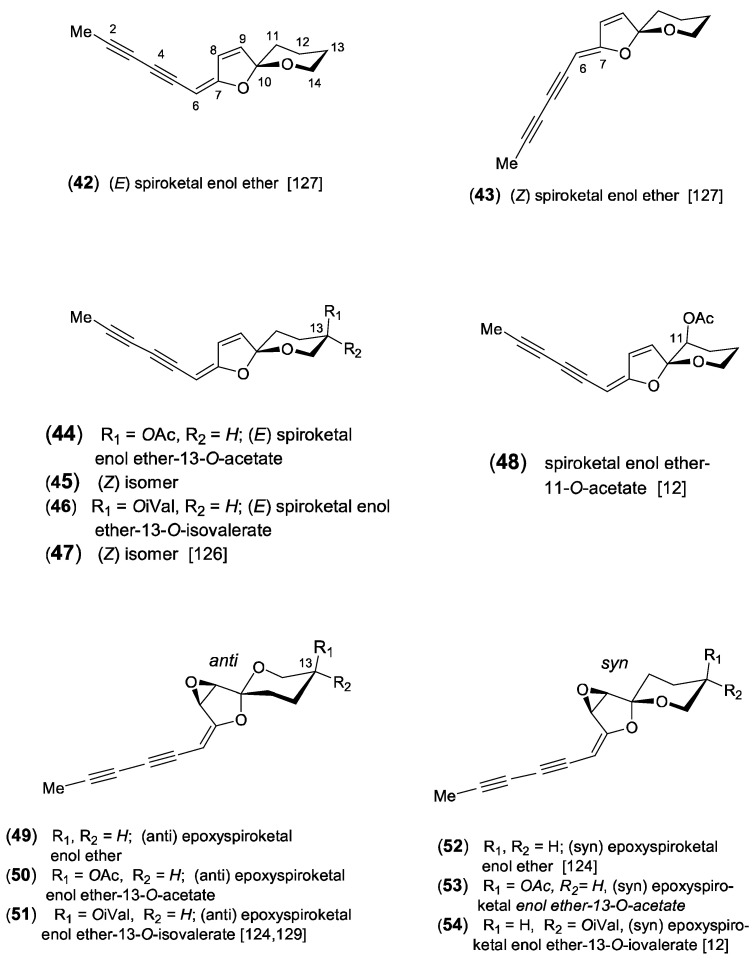

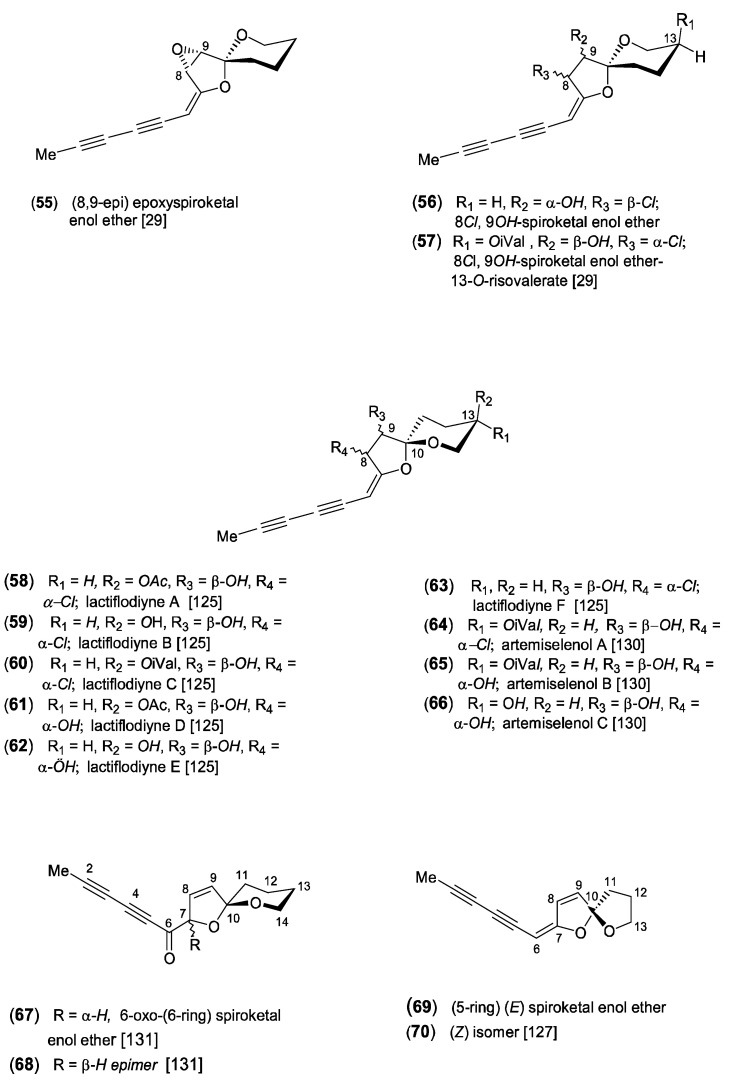

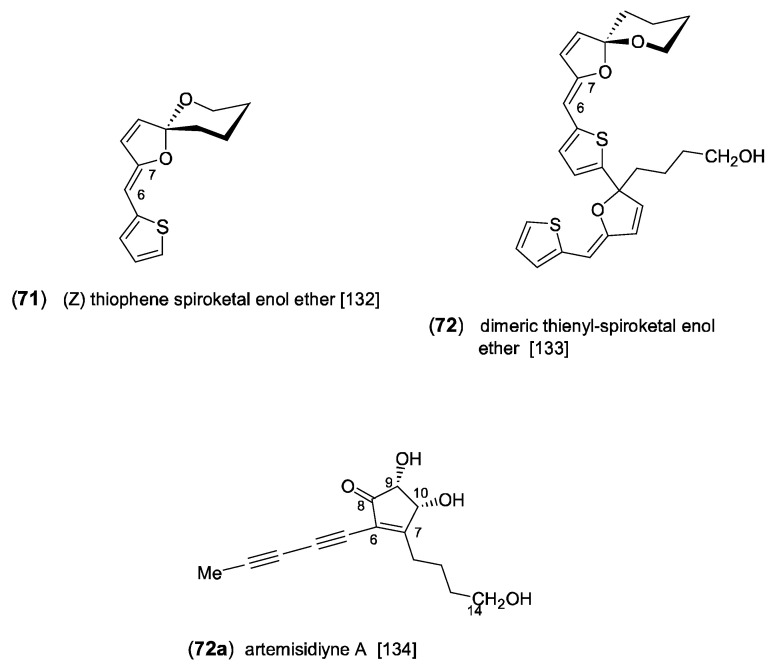
Spiroketal enol ethers [12,29,124,125,126,127,129,130,131,132,133,134].

**Scheme 4:**
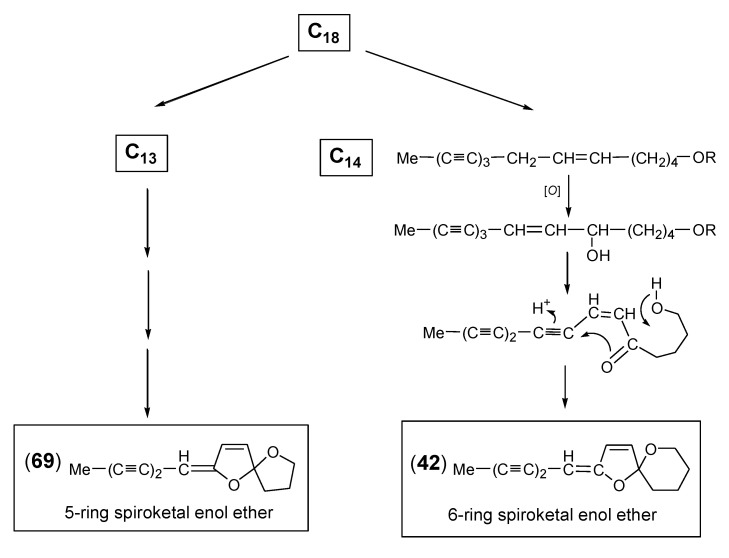
Biosynthesis of 5-ring (**69**) and 6-ring (**42**) spiroketal enol ether.

The formation of spiroketal enol ethers represents a typical chemical character of the tribe Anthemideae and plays a dominant role in the acetylenic profiles of many *Artemisia* species [7,9,13]. Biogenetically, the bicyclic structure is derived from a linear triyne-ene alcohol and is transformed via a ketoalcohol intermediate [122,123] (Scheme 4). Due to their characteristic UV spectra with only weakly defined broad maxima, they can be clearly distinguished from other acetylenes (graphics in [1] and especially in [13]). They were isolated either as *C*_14_-six- or *C*_13_-five-membered ringenol ethers with stereochemically complex structures [124,125]. Both were reported to co-occur in *A. pedemontana* Balb., where the 6-ringenol ethers dominated in the aerial parts and the 5-ringenols in the roots [126]. In the first report on structure elucidation, Bohlmann et al. [127] described the isolation of the (*E*)-configurated 6-ringenol ether (**42**) from *Tanacetum vulgare* L. and the corresponding 5-ringenol ether (**69**) from *Matricaria discoidea* DC. (syn.: *M*. *matricarioides*) together with the (*Z*)-isomers (**43**) and (**70**), respectively. Structural variation is created by ester groups mostly attached at position *C*-13 of the six-membered oxane ring (Figure 7). The (*E*)-acetate (**44**) and the (*E*)-isovalerate (**46**) were isolated from *A. pedemontana* along with small amounts of the (*Z*)-isomers (**45**) and (**47**). All ester groups were shown to be uniformly attached at *C*-13 in axial orientation [126]. Further modification was reported for *A. caruthii* Wood with an acetoxy group attached at the *C*-11 position (**48**) [12].

Additional oxidation leads to the formation of tricyclic epoxides of spiroketal enol ethers [128]. From *A. douglasiana* Bess., the epoxide of (*E*)-6-ringenol ether (**49**) was isolated, along with the corresponding acetate (**50**) and isovalerate ester (**51**). However, the relative configuration of the epoxide ring was not determined [129]. A series of related, but chromatographically deviating, epoxides was later detected in *A. selengensis* Turcz. Based on detailed stereochemical analysis, it was shown that the 6-ringenol ether (**52**) represented a novel type of stereoisomers characterized by a “*syn*” arrangement of the epoxide ring relative to the oxygen of the six-membered oxane ring [124]. This relative configuration could also be confirmed for the co-occurring acetate (**53**) and isovalerate ester (**54**) but is in contrast to the “*anti*” configurated epoxides (**48–50**) found in *A. douglasiana* and other species investigated so far. The isovalerate group of (**54**) deviated by an equatorial orientation [12]. Ten years later, a new epimer (**55**) was detected in the leaves of *A. lactiflora* Wall. ex DC., along with three known epoxides (**49–51**) and the new chlorohydrins (**56**) and (**57**) [29]. In a reinvestigation of *A. lactiflora*, six closely related derivatives, named lactiflodiynes A-F (**58–63**), were isolated and showed different substitutions at *C*-8 and *C*-9 (Figure 7). Their structures were elucidated by extensive spectroscopic methods, X-ray crystallography, chemical transformations, and CD. The absolute configuration of lactiflodiyne A (**58**) was determined to be 8R, 9S, 10S, and 13R. It was shown that derivatives with an unsubstituted *C*-13 position contained a mixture of both 10R and 10S analogs in the same plant, while in those with a substituted *C*-13, only the 10S configuration was identified [125]. In addition, three further derivatives were isolated from *A. selengensis*, named artemiselenols A-C (**64–66**). The absolute configuration of artemiselenol A (**64**) was determined by X-ray crystallography to be 8R, 9S, 10S, and 13S [130]. The inclusion of oxygen at *C*-6 leads to the two epimers (**67**) and (**68**) of a keto-6-ringenol ether, isolated from the leaves of *A. feddei* H.Lév. &Vanot [131]. An important structural modification of spioketal enol ethers is created by the incorporation of sulfur, leading to the formation of thiophenes. The thienyl-substituted spiroketal (**71**) was isolated from the North American *A. ludoviciana* Nutt. [132]. It was shown to represent the major constituent in several other *Artemisia* species accompanied by the dimeric derivative (**72**) [13,133]. From the aerial parts of *A. lactiflora* (10 kg), a novel *C*_14_-diacetylenic compound was isolated, named artemisidiyne A (**72a**) [134]. Its unprecedented skeleton allows the expectation of biogenetic connections to the spiroketal-type pathway (Figure 7).

### 2.4. Capillen-Isocoumarin Type

**Figure 8:**
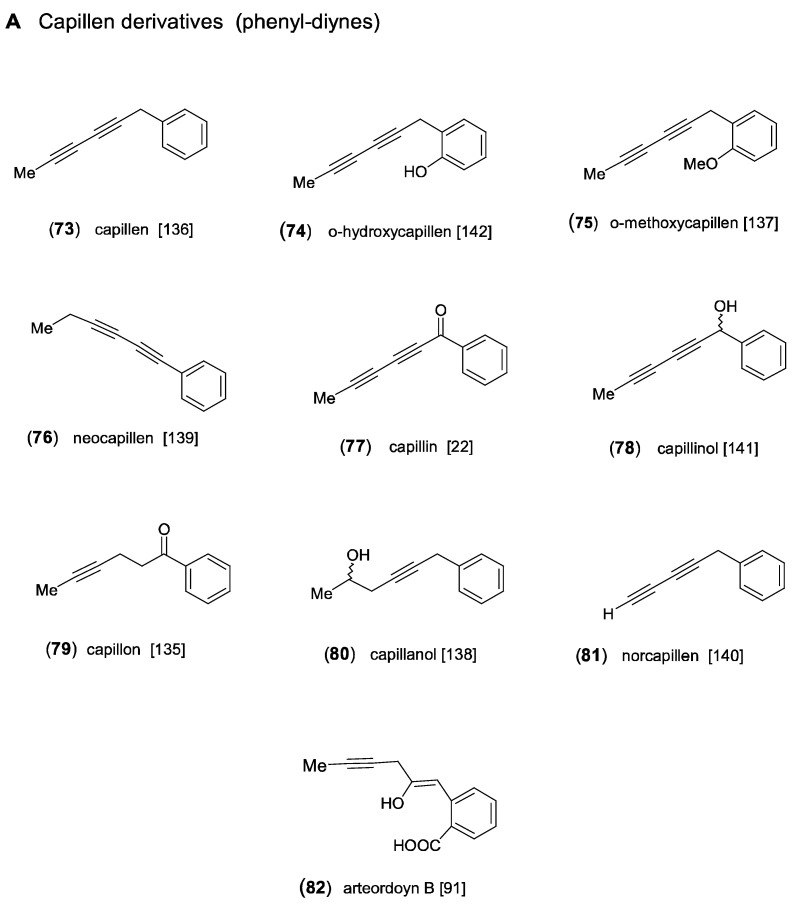

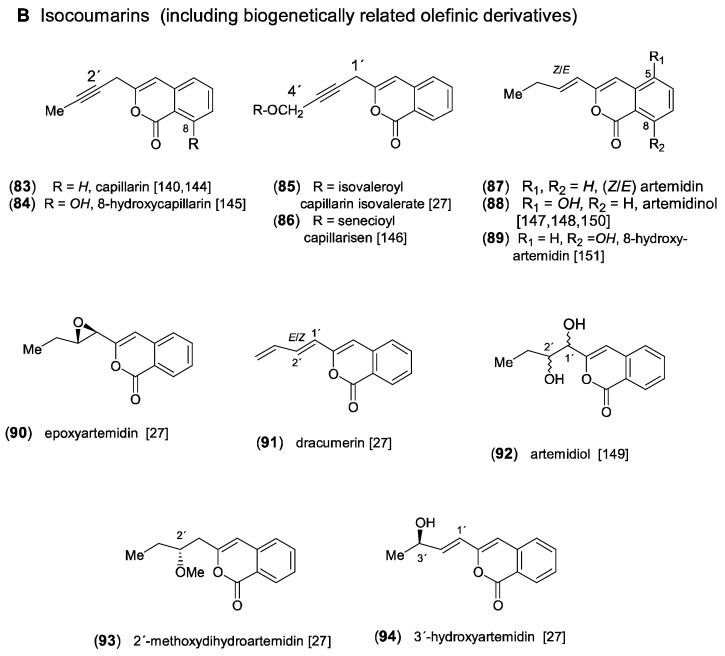
Aromatic acetylenes and isocoumarins [22,27,91,135,136,137,138,139,140,141,142,144,145,146,147,148,149,150,151].

**Scheme 5:**
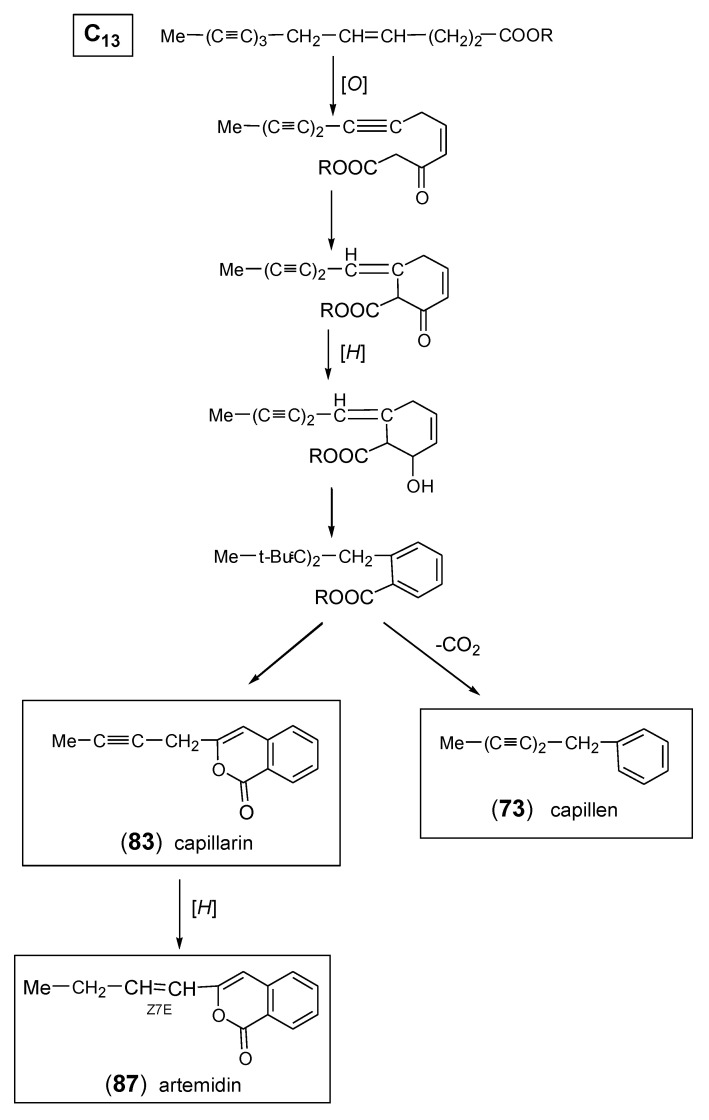
Biosynthesis of aromatic capillen (**73**) and isocoumarin (capillarin) (**83**).

Feeding experiments have shown that all aromatic acetylenes detected in *Artemisia* and in the tribe Anthemideae are derived from a linear *C*_13_-triyne-enoic precursor. As demonstrated in Scheme 5, the most important biosynthetic steps were proposed to be β-oxidation followed by a “Michael addition” leading to a cyclized ketoester, which is further converted to an aromatic intermediate. This aromatic ester represents the precursor of both the phenyl-diynes and the isocoumarins (Figure 8) [1,14]. The first aromatic acetylenes were isolated from the essential oil of *A. capillaris* Thunb. and were identified as the phenyl-diynes capillen (**73**) [135] and capillin (**77**) [22]. In following GC/MS analyses of *A. capillaris*, the related derivatives capillon (**79**) [136], neocapillen (**76**), capillanol (**80**), and *o*-methoxycapillen (**75**) were also detected, from which (**76**) was supposed to be an artifact of (**73**) created by UV-irradiation [137,138,139]. Neocapillen (**81**), characterized by an acetylenic end group, was isolated from the lipophilic root extract of *A. dracunculus* L. [140]. In addition, capillinol (**78**) was later obtained from *A. capillaris* collected in Korea [141]. From the aerial parts of *A. ordosica* Krasch., *o*-hydroxycapillen (**74**) was isolated, together with arteordoyin B (**82**) [91]. Compound (**74**) was originally isolated from *Chamaemelum fuscatum* (Brot.) Vasc. (=*Anthemis fuscata* Brot.), and its structure was elucidated by Bohlmann and Zdero [142]. The structure of arteordoyin B (**82**) (Figure 8) with an aromatic carboxy group and an enol functionality in the side chain can be regarded as a biogenetic precursor of isocoumarins (Scheme 5) [91].

The formation of isocoumarins is an important biosynthetic step leading to major constituents in *Artemisia* species of the subgenus Dracunculus [7,11,27,95,143]. The acetylenic isocoumarin capillarin (**83**) was first isolated from *A. capillaris* [144] and was later also detected in *A. dracunculus* [140]. Structural variation is created by hydroxylation of the aromatic ring in 8-hydroxycapillarin (**84**) [145] and of the side chain, where it forms an ester with isovaleric acid (**85**) in *A. dracunculus* [27] and with senecioic acid (**86**) in *A. arctica* Less., named capillarisen [146]. Another type of isocoumarin was also found in *A. dracunculus*, differing by an olefinic side chain. The corresponding derivatives were named artemidin (**87**), artemidinol (**88**), and artemidiol (**92**) [147,148,149]. Artemidin (**87**) was also reported independently for *Chamaemelum fuscatum* (Brot.) Vasc., where it was shown to coexist with the acetylenic capillarin (**83**), indicating close biogenetic connections [150]. In a reinvestigation of *A. dracunculus*, originating from Kyrgyzstan, the olefinic isocoumarins (*Z*/*E*)-artemidin (**87**), artemidinol (**88**), and the new 8-hydroxyartemidin (**89**) were isolated as major constituents [7]. As shown in a following chromatographic comparison of lipophilic root extracts of the taxonomically complex *A. dracuncus* group, different accumulation trends towards phenyl-diynes, acetylenic or olefinic isocoumarins represent significant chemical characters [11]. Additional structural variation of the olefinic side chains of artemidins leads to the formation of epoxyartemidin (**90**), dracumerin (**91**), 2′-methoxydihydroartemidin (**93**), and 3′-hydroxyartemidin (**94**) [27].

## 3. Biological Activities

### 3.1. Antifungal Activity

The discovery of the antifungal activity of safynol, a *C*_13_-acetylene from safflower (*Carthamus tinctorius* L.; Asteraceae) [64], and falcarindiol, a *C*_17_-acetylene from *Aegopodium podagraria* L.(Apiaceae) [37], awakened the biological interest in this class of compounds. This was further intensified by the exploration of the mode of action of falcarindiol against *Mycocentrospora acerina* (Hartig) Deighton, a storage pathogen of carrot (*Daucus carota* L.). Here, it was shown that 28 µg/mL burst bimolecular lipid membranes formed from lecithin or lecithin-cholesterol and caused hemolysis in erythrocytes [38,39]. The protective role of falcarinol and falcarindiol was made apparent by their formation as phytoalexins in tomato leaves (*Lycopersicon esculentum* Mill.) [40]. This response to a fungal attack in a member of the Solanaceae, a family normally without polyacetylenes, impressively underlined the function of acetylene accumulation as a defensive mechanism. Antifungal activity was later also reported for the structurally closely related dehydrofalcarinol (**1**) and dehydrofalcarindiol (**3**), isolated from *Artemisia borealis* Pallas [42,43]. Since their structures differ only by an additional isolated double bond, similar molecular mechanisms may be expected. A more recent study provided information about the antifungal properties of six derivatives (**1, 3**, **8**, **9**, **12**, **15**); (Figure 2). They were isolated from *A. halodendron* Turcz. ex Bess. and tested against five different fungi. The highest values for mycelial growth inhibition were reported for the 3R,10S-configurated *C*_17–_1,8,16-triene-4,6-diyne-3,10-diol, named artehaloyn A (**8**), and for 3*R*,8*S*-dehydrofalcarindiol (**3**) (=artehaloyn B) against the two fungi *Cladosporium curcumerinum* and *Magnaporthe oryzae*. The antifungal values were comparable with those of the commercial fungicide carbendazin [46].

A second group of antifungal acetylenes is represented by derivatives of the aromatic capillen-isocoumarin type (Figure 1 and Figure 8). The first active compound was isolated from *A. capillaris* and named capillin (**77**) [22]. Two further antifungal derivatives, norcapillen (**81**) and capillarin (**83**), were obtained from the essential oil fraction of *A. dracunculus* L. They were detected by bioautography on TLC plates and tested against *Colletotrichum fragariae*, *C. gloeosporioides*, *C. acutatum*, and *Botrytis cinerea*. The isocoumarin capillarin (**83**) was shown to be more effective, especially against *B. cinerea*, where 30 µmol caused 60% growth inhibition [152]. A more detailed analysis of 13 naturally occurring and 11 synthetic isocoumarins (including acetylenic and biosynthetically related olefinic derivatives) provided information about structure–activity relationships in this group of compounds. It was shown that the structural variation of the butyl side chain plays an important role in different activities [27]. The mechanism of action of the antifungal phenyl-heptatriyne was interpreted as a disruption of membranes, enhanced in the presence of sunlight [26]. Regarding the similar molecular structures of capillen derivatives, it is tempting to assume similar mechanisms.

The antifungal activity reported for *A. absinthium* L. (“wormwood”) [109] should be reconsidered, since the structures of the active *C*_12_-dithiophenes apparently do not fit in with the chemical makeup of the genus *Artemisia* (Figure 1) or the tribe Anthemideae. Antifungal activity was also reported for spiroketal enol ethers isolated from *Dendranthema zawadskii* (Maxim.) Kitam. (tribe Anthemideae), which were tested against the human-pathogenic fungus *Trichophyton mentagrophytes* [34]. However, in the genus *Artemisia*, antifungal activities are solely established so far for derivatives of the dehydrofalcarinol and aromatic capillen-isocoumarin type (Figure 1).

### 3.2. Insecticidal Activity

On the basis of leaf-disk choice tests, Yano [25] described the insect antifeeding properties of the essential oil of growing buds of *A. capillaris* against larvae of *Pieris rapae* (“cabbage white butterfly”). It was shown that the two aromatic acetylenes capillen (**73**) and norcapillen (**81**) were responsible for activity. In this connection, a possible ecological role of aromatic acetylenes was proposed for their different accumulation in two chemotypes of *A*. *capillaris* in Japan, collected near a river bank and at the sea shore. While the roots of both were characterized by compounds (**73**) and (**76**), the aerial parts clearly differed by accumulating acetylenes only in plants from the river bank [153]. Follow-up studies indicated that both the presence and different positions of triple bonds in the side chain influenced activity and intensity [154,155]. Another aromatic acetylene with insecticidal properties was reported for *A. monosperna* Del. [156]. However, the published structure was assumed to be incorrect [77,97]. Most likely, it represented capillen (**73**) again. In the search for naturally occurring agrochemicals, the essential oil of *A.ordosica* Krasch. was shown to possess repellent and fumigant activity against *Tribolium castaneum* (“flour beetle”). The active components were identified as the aromatic acetylenes (**73**, **76**, **77**), together with (*Z*)-DME (**21**) [157].

In addition to aromatic acetylenes, insecticidal properties were also reported for derivatives of the dehydrofalcarinol type. While a preliminary screening already exhibited larvicidal activity of dehydrofalcarinol (**1**) and dehydrofalcarindiol (**3**) against *Aedes aegypti* (“yellow fever mosquito”) [42,43], a more detailed test of six different derivatives (**1**, **3**, **8**, **9**, **12**, **15**) (Figure 2) provided information about structure–activity relationships. They were isolated from *A*. *halodendron* and tested against *Bradysia odoriphaga* (“chive gnat”; Diptera). While the *C*_17_-derivatives (**1**, **3**, **8**, **9**) showed even higher values than the control azadirachtin, the *C*_10_-derivatives (**12**, **15**) were clearly less toxic. Among the active compounds, dehydrofalcarinol (**1**) was the most effective, followed by artehaloyn A (**8**) [46].

Insecticidal activity was also reported for acetylenes of the spiroketal enol ether type [31,35,158]. In the genus *Artemisia*, three derivatives (**42**, **43**, **69**), isolated from aerial parts and roots of *A. granatensis* Boiss., showed antifeeding effects against the three pest insects *Spodoptera littoralis*, *Rhopalosiphium padi*, and *Myzus persicae* [159]. Moreover, insecticidal activity is also known from pontica epoxide (PE) (**40**) [160] and (*Z*)-DME (**21**) [157], representing derivatives of the linear triyne type (Figure 1).

### 3.3. Nematicidal Activity

Apart from the well-known nematicidal, acetylene-derived, thiophenes from *Tagetes* species (“marigold”) [50,66], another type of nematicidal dithiophenes was reported for *A. absinthium* [109]. However, as already mentioned in Section 2.3 (DME), the identity of the plant material should be reconsidered, since this structural type of acetylenes is unknown from the genus *Artemisia* as well as from the tribe Anthemideae. Polyacetylenes with nematicidal properties were first isolated from *Carthamus tinctorius* L. (Asteraceae) and identified as (*E*/*Z*)-isomers of *C*_13_-ene-triyne-ene [161,162]. Significant nematicidal activity was later reported for the two *C*_17_-acetylenes falcarinol and falcarindiol, isolated from *Hansenia weberbaueriana* (Fedde ex H. Wolff) Pimenov & Kijuykov (Apiaceae) [51]. From the lipophilic extract of *A. halodendron*, six derivatives of the structurally related dehydrofalcarinol type (**1**, **3**, **8**, **9**, **12**, **15**) (Figure 2) were shown to exhibit remarkable effects against *Meloidogyne incognita* (“root-knot nematode”). Among the active compounds, artehaloyn A (**8**) showed the highest LC_50_ value (0.21 ± 0.03 mg/L), which was much higher than that of the control abamectin [46].

### 3.4. Cytotoxic Effects

Cytotoxic acetylenes were first described for *Panax giinseng* C.A. Meyer (Araliaceae) and were identified as *C*_17_-falcarinol (=panaxynol) derivatives [73]. Based on experiments with living membranes and artificial lipid bilayers, the cytotoxic effect was interpreted as a result of membrane damage [163]. Tests with different cancer cells exhibited a much higher growth-inhibitory effect against malignant cells than against normal cells [52]. The high cytotoxicity of falcarinol-type acetylenes was speculated to be associated with their ability to form extremely stable carbocations, thereby acting as very reactive alkylating agents toward biomolecules [20,60]. The structurally corresponding dehydrofalcarindiol (**3**), isolated from *Dendropanax arboreus* (L.) Decne & Planch. (Araliaceae), was tested against four different tumor cell lines (Hep-G2, A-431, H-4IIE, L-1210) at 0.025% (*w*/*v*) and showed 100% killing in all cell lines [55]. The comparison of cytotoxicities between three dehydrofalcarinols (**1**, **3**, **8**) and those of the corresponding falcarinols, coexisting in *D. arboreus,* showed that the inclusion of the characteristic vinyl end group led to a slight reduction in potencies [56]. In a follow-up study, the 3R,8R-stereoisomer of dehydrofalcarindiol (**3**), isolated from *A. monosperma* Del., was tested against four colorectal (LS174T, SKOLO1, COLO320DM, WIDR) and two breast cancer cell lines (MDA231, MCF7). The greatest efficacy was determined for dehydrofalcarindiol (**3**) with IC_50_ values at 5.8 µg/mL against MCF7 and 9.6 µg/mL against COLO320DM. Interestingly, this was the opposite of how the latter cell line responded to the cytostatic doxorubicin, where it was highly resistant [57]. The pronounced antitumor activity of dehydrofalcarindiol (**3**) was also reported for the root extract of *Gymnaster koraiensis* (Nakai) Kitam. (Asteraceae) tested against L1210 mouse leukemia cells. Among eight different falcarinol-related *C*_17_-derivatives, including gymnasterkoreaynes A-F, dehydrofalcarindiol (**3**) clearly showed the highest value with an ED_50_ at 0.12 µg/mL [164]. Apart from these active *C*_17_-compounds a new type of *C*_14_-diacetylene, named artemisidiyne A (**72a**), was isolated from *A. lactiflora* and showed cytotoxic activity against HCT-8, BGC-823, and A 549 tumor cell lines with IC_50_ values at 7.5, 1.1, and 4.6 µmol, respectively [134].

Whelan and Ryan [28] investigated the effects of the aromatic acetylene capillin (**77**) on four human tumor cell lines to examine both its anti-proliferative and pro-apoptotic properties. HT 29 (colon), MIA PaCa-2 (pancreatic), HEp-2 (epidermoid of larynx), and A549 (lung) tumor cells were used, and capillin (**77**) inhibited their viability in a dose- and time-dependent manner. It was most efficacious against HEp-2 cells with IC_50_ values at 2.8 µmol (24 h), 0.8 µmol (48 h), and 0.6 µmol (72 h). Around 25% of all tested cells displayed cell shrinkage and loss of cell-to-cell contact after treatment with 1–8 µmol capillin (**77**). Apart from observations with fluorescence microscopy, apoptotic cell death was additionally confirmed by flow cytometry and agarose gel electrophoresis. Capillin also caused the accumulation of cells in the S + G_2_/M-phase of the cell cycle, increased glutathione levels in tumor cells, and inhibited the synthesis of macromolecules (DNA, RNA, proteins). The potent apoptosis-inducing activity of capillin was later also confirmed by using human leukemia HL-60 cells. In this investigation, it was shown that capillin (**77**) was more potent than capillen (**73**), and cell death was induced via the mitochondrial pathway [165].

### 3.5. Allelopathic Effects

The *C*_10_-acetylenic ester DME (**21**) and the related lactone **23** of the linear triyne type (Figure 4) were shown to strongly inhibit the growth of seedlings of the widespread weed *Echinochloa crus-galli* (L.) P.Beauv. (“barnyard millet”). DME (**21**), detected in many *Artemisia* species, was investigated in *Solidago altissima* L., where its allelopathic effect was regarded as one of the factors of the great propagating potency of this plant [69]. In a follow-up study, the two isomers **21** and **22** were found in the soil at the border of *S. altissima* communities in concentrations that were inhibitory to the test plants [70]. Inhibition of seed germination of six different plants was described for the aromatic polyacetylene capillen (**73**) isolated from the roots of *A. capillaris*. In this test, capillen (**73**) completely inhibited the germination of the four plants *Setaria italica*, *Brassica oleracea*, *Viola tricolor*, and *Daucus carota*. It showed only weak inhibition against *Chrysanthemum coronarium*, but no effect against *Medicago sativa* [71].

### 3.6. Miscellaneous Properties

#### 3.6.1. Antibacterial Activity

Apart from the *C*_10_-4,6-diynoic acid and its methyl ester, isolated from *Bellis perennis* L. (Asteraceae) [166], antibacterial acetylenes are mainly known from the family Apiaceae. Here, they were represented by *C*_17_-derivatives of the falcarinol type [44,53,54]. In the genus *Artemisia*, the *C*_13_-acetylene pontica epoxide (PE) (**40**) from the linear triyne type (Figure 6) was shown to significantly contribute to the antibacterial activity of the lipophilic extract of the aerial parts of *A. annua* L. It was tested against the Gram-positive *Clostridium perfringens*, the causative agent of necrotic enteritis, which leads to severe losses in the poultry industry [120].

#### 3.6.2. Antiviral Activity

Only scattered information is available on polyacetylenes with antiviral properties. Based on bioassay-guided fractionation, significant activity against *Herpes simplex virus* type 1 (HSV-1) and 2 (HSV-2) was detected for spiroketal enol ether (**42**), a major constituent of many *Artemisia* species. HSV-1 and HSV-2 are enveloped viruses, characterized by a large linear double-stranded DNA genome, and are major human pathogens. In the case of the spiroketal (**42**), it was shown that adsorption inhibition and viricidal activity appeared to be not relevant. Instead, the most significant effects were the inhibition of virus penetration and a novel mechanism consisting of the specific arrest of viral gene expression and, consequently, the decrease in viral protein accumulation within infected cells [36]. Antiviral activity was also reported for the *C*_10_-dehydrofalcarinol-type acetylenes (**13a**) and (**13b**) against *Hepatitis B virus* (HBV). They were isolated from the aerial parts of *A. scoparia* Waldst. et Kit. and were shown to represent rare caffeoyl-substituted glucosides [94]. However, since antiviral activity was also detected in other coexisting caffeoylated compounds without acetylenic structures [93], it can be assumed that caffeoyl substitution is mainly responsible for the activity here.

#### 3.6.3. Inhibition of Superoxide Generation

**Figure 9:**
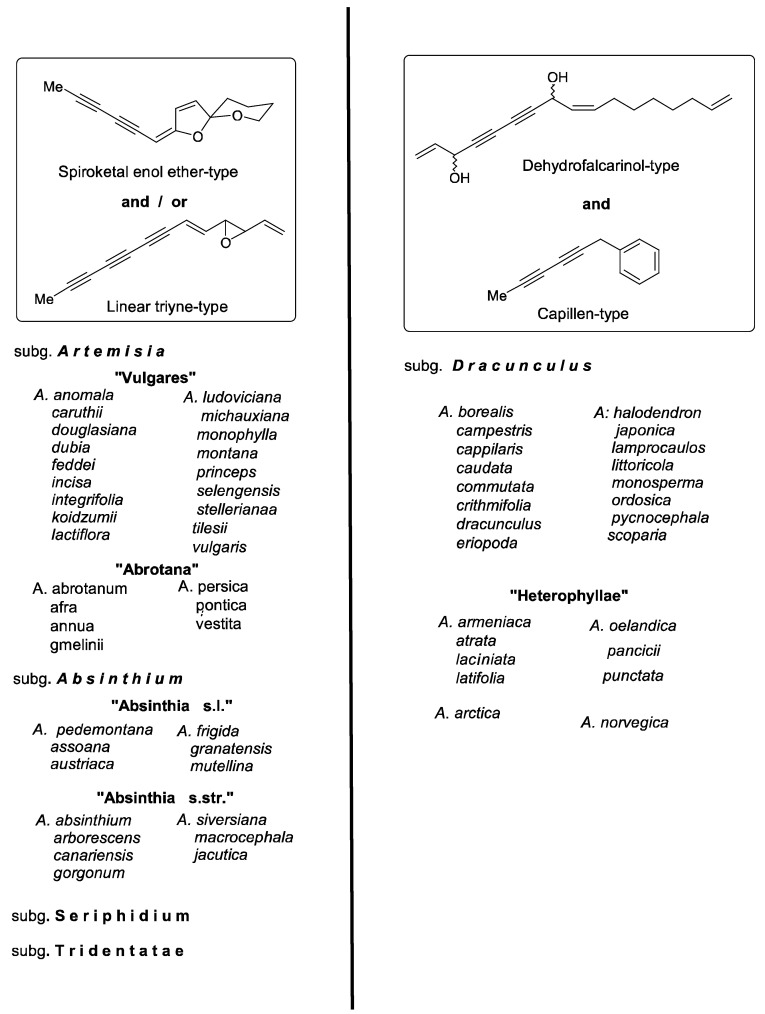
Infrageneric grouping of *Artemisia* based on the formation of characteristic structural types of polyacetylenes.

The leaf extract of *A. lactiflora* Wall. ex DC. (Figure 9), an edible species from Southeast Asia, was shown to inhibit PMA (phorbol myristate acetate)-induced superoxide (*O*_2_^−^) generation. A group of spiroketal enol ethers were identified as active constituents, from which the epoxide of the isovalerate ester (**51**) was an especially potent inhibitor with an IC_50_ value at 7.6 µmol. In contrast, the activities of the closely related derivatives (**49**) and (**55**) (Figure 7) with IC_50_ = 47 µmol and 43 µmol, respectively, were much weaker. The results provided information about structure–activity relationships and suggested that an acyloxy group at the *C*-13 position enhanced inhibition, whereas the absolute configurations are not important [29]. In a follow-up study, the authors confirmed the inhibition effects of spiroketal (**51**) on a variety of tumor-promoter-induced biological responses, such as oxidative stress, as well as tumor promotion in ICR mouse skin [30].

#### 3.6.4. Anti-Inflammatory Activity

The anti-inflammatory activity of acetylenes was first reported for *Daucus carota* L., where it was investigated by determining the attenuation of the response to LPS (lipopolysaccharide) induction. The isolated *C*_17_-falcarinol derivatives reduced nitric oxide (NO) production in macrophage cells by as much as 65% without cytotoxicity [167]. In *A. halodendron*, the activity was attributed to the two dehydrofalcarinol derivatives (**3**) and (**9**) (Figure 2). They inhibited the levels of NO, TNR-α (tumor necrosis factor), and IL-6 (interleukin) in LPS-induced RAW 264.7 cells in a dose-dependent manner [92].

#### 3.6.5. Inhibition of TGF-β1-Induced Liver Cell Apoptosis

Experiments with transforming growth factor (TGF)-β1-induced apoptosis in hepatocytes exhibited a strong inhibitory effect of the aromatic acetylenes capillin (**77**) and capillen (**73**). They were isolated from *A. capillaris* as part of the Japanese herbal medicine “inchin-ko-to”. The inhibition of undesired apoptosis induced by TGF-β1 was expected to be beneficial for the treatment of various inflammatory liver diseases [168].

#### 3.6.6. Anti-Diabetic Potential

The anti-diabetic potential of the two aromatic acetylenes capillin (**77**) and capillinol (**78**), isolated from *A. capillaris*, was investigated by testing the ability to inhibit the enzymes α-gucosidase, protein tyrosine phosphatase 1B (PTP1B), and rat lens aldose reductase (RLAR). In particular, capillin (**77**) showed potent inhibitory activity with an IC_50_ value at 332.96 ± 1.44 µmol against α-gucosidase, similar to the positive control acarbose with IC_50_ = 320.33 ± 4.61 µmol. In contrast, the efficacy of capillinol (**78**) against α-gucosidase and PTP1B was clearly weaker and showed even no activity against RLAR, suggesting that the keto group of capillin is the major determinant of its anti-diabetic potential [141].

## 4. Conclusive Remarks

The infrageneric classification of *Artemisia* is mainly based on floral characters and DNA-sequence data. According to that, the species are traditionally separated into the five subgenera *Artemisia*, *Absinthium*, *Seriphidium*, *Dracunculus*, and *Tridentatae*. However, as pointed out in a comprehensive treatise of the biology of the genus, many problems remained to be solved regarding a more natural taxonomic arrangement both at the subgeneric as well as the species level [169]. Apart from palynological and cytogenetic results, promising contributions were also expected from phytochemical data. Stimulated by pharmaceutical interests, most studies focused on the distribution and activity of sesquiterpene lactones and monoterpenes, while other typical classes of compounds, such as flavonoids, coumarins, lignans, and polyacetylenes, were less intensely investigated [77]. Due to the typical UV spectra of polyacetylenes, broad-based UV-HPLC comparisons of lipophilic crude extracts of a representative set of *Artemisia* species provided information about the distribution of characteristic derivatives. Together with leaf and floral characters, they were shown to represent chemical markers that corresponded remarkably well with the classical infrageneric classification [7,9,10,11,13]. Considering the biogenetic origin, their structures were grouped into the (a) dehydrofalcarinol, (b) capillen-isocoumarin, (c) spiroketal enol ether, and (d) linear triyne type (Figure 1). One of the most striking chemical differences within the genus was the vicarious occurrence between types (a) and (b) in the subgenus *Dracunculus* and types (c) and (d) in the other subgenera (Figure 9).

Within the subgenus *Dracunculus*, various aromatic acetylenes were shown to characterize different samples of *A. dracunculus* itself. In addition to dehydrofalcarinol derivatives, the diploid representatives, published as *A*. *dracunculiformis*, *A. glauca*, and *A. pamirica*, mainly accumulated capillen (**73**), while the polyploids of *A. dracunculs* s.str. differed by isocoumarins (Figure 8). The formation of capillarin (**83**) distinguished the decaploid sample from hexa- and octoploids, originating from central Asia, which mainly contained olefinic isocoumarins (**87–89**) [11]. The co-occurrence of structural types (a) and (b) was also shown to be typical for the taxonomically complex *A. campestris*–*A. capillaris* group. More detailed investigations revealed structural diversification of dehydrofalcarinol derivatives by mid-chain oxidation and shortening to *C*_10_-derivatives. They were isolated from the aerial parts of *A. eriopoda* Bunge [98] and *A. halodendron* [46]. The formation of structurally corresponding *C*_10_-glucosides (Figure 2) in *A. monosperma* [57], *A. capillaris* [93], and *A. scoparia* [94] underlined the common biogenetic trend. Considering this clear chemical segregation, the additional occurrence of dehydromatricaria ester (DME) (**21**) of the linear triyne type in *A. ordosica* Krasch. is noteworthy [91,157].

Of particular chemotaxonomic interest is the predominance of dehydrofalcarinols in the “Heterophyllae” and “Norvegicae”, two species groups placed in the subgenus *Artemisia* on the basis of morphological characters. A close phylogenetic relationship between the “Heterophyllae” and *A. norvegica* Fries. and *A. arctica* Less. was suggested by floral and cytological data [170]. The formation of dehydrofalcarinone (**2**) in *A. norvegica* (collected in Dovrefjell, Norway; Greger unpubl.) and the co-existence of dehydrofalcarinol (**1**) with the aromatic acetylenes capillen (**73**), capillarin (**83**), and capillarisen (**86**) in *A. arctica* strengthened this proposal [146]. The detection of dehydrofalcarinone (**2**) in the taxonomically rather isolated East Asiatic *A. keiskeana* Miq. suggested a relationship with the subgenus *Dracunculus* [9].

The formation of spiroketal enol ethers is a prominent chemical feature of the subgenera *Artemisia* and *Absinthium* (Figure 9). In spite of infraspecific variation, leading to a lack of spiroketals in European samples of *A. vulgaris* L. or within *A. princeps* Pamp., originating from Mt. Aso in Japan, they represent a basic biogenetic trend of the “Vulgares” group within the subgenus *Artemisia* [13]. Structural variation was created by different ester groups, epoxidations, stereochemistries, and incorporation of sulfur. However, only *C*_14_-derivatives of six-membered ringenol ethers have been reported so far. By contrast, in the subgenus *Absinthium*, the co-existence of *C*_13_-five-membered ringenols (Scheme 4) was detected in *A. pedemontana* Balb. [127] and in the alpine *A. mutelina* Vill. group [1,171]. This biogenetic trend was later also confirmed for *A. austriaca* Jacq. and *A. frigida* Willd. [10,172], as well as for *A. assoana* Willk. [173] and *A*. *granatensis* Boiss. [159]. In view of these results, it appears of taxonomic relevance that in *A. absinthium* L. itself, and in the probably closely related shrubby members *A. arborescens* L. and *A. canariensis* Less., only the (*Z*)-isomers of 6-ringenol ethers (published as “trans”-configurated) were detected [10]. The relationship of this species group, including *A. gorgonum* Webb., *A. siversiana* Ehrh. ex Willd., *A. macrocephala* Jacqem. ex Bess., and *A. jacutca* Drob., was additionally confirmed by an accumulation of sesamin-type lignans [9,172,174,175,176]. With the accumulation of the lignan syringaresinol together with the 6-ringenol ether and its thiophene derivative, the Northwest African *A. reptans* C. Sm. [114] also suggested relations with this species group.

Apart from spiroketal enol ethers, the two subgenera *Artemisia* and *Absinthium* are additionally characterized by acetylenes of the linear triyne type (Figure 1 and Figure 9). Within the subgenus *Artemisia* a group of species deviates by a predominance of the “triyne-triene” (**38**) and particularly ponica epoxide (PE) (**40**), linked with a lack of spiroketals. This group, informally named “Abrotana”, includes *A. abrotanum* L., *A. afra Jacq.* [115], *A. persica* Boiss., *A. pontica* L. [116], and *A. annua L.* [119]. With the exception of *A. persica* they were previously placed in the section *Abrotanum*. The relationship of the “Abrotana” was also suggested by the formation of characteristic sesquiterpene-coumarin ethers [9,177].

Only scattered information is available on polyacetylenes of the subgenera *Seriphidium* and *Tridentatae*. As already pointed out previously, all Eurasiatic members of *Seriphidium* were characterized by a general reduction of acetylenes, comprising linear triynes combined with a lack of spiroketals, while in the North American *Tridentatae*, only small amounts of spiroketals were observed. They were detected in preliminary analyses due to their characteristic UV spectra [9]. The biogenetic trend of subg. *Seriphidium* was later confirmed with the isolation and identification of the linear triynes centaur X_3_ (**16**), “triyne-triene” (**38**), and PE (**40**) from the roots of *A. santonicum* L. ssp. *patens* (Neilr.) Person, collected in East Austria (Bohlmann and Greger, unpubl.). These derivatives resembled those found in the subgenus *Artemisia*. In this connection, it should be pointed out that the report on spiroketal enol ethers and sesamin-type lignans in *A*. *fragrans* Willd., a member of the subgenus Seriphidium [178], has obviously been based on misidentified plant material. Most likely, it was confused with *A. absinthium* [175].

As demonstrated in Figure 9, acetylenes of the dehydrofalcarinol type play an important role in the infrageneric classification of *Artemisia*. They not only support the taxonomic segregation of the subgenus *Dracunculus*, but also represent a significant chemical character of the “Heterophyllae” group, previously placed in subgenus *Artemisia* [170]. With respect to the formation of dehydrofalcarinols in the isolated, south-hemispheric Anthemideae genera *Eriocephalus* and *Coutla*, as well as their close structural relationship to falcarinols, dominating in the families Pittosporaceae, Araliaceae, and Apiaceae, this biogenetic trend is of special phylogenetic interest. In *Artemisia*, this type of acetylenes was shown to be specifically linked to the formation of aromatic acetylenes of the capillen-isocoumarin type (Figure 1). This differs from other findings in the tribe Anthemideae, where in the *Chrysanthemum* group, aromatic acetylenes coexist with spiroketal enol ethers [1], and in the genus *Chamaemelum* (published as *Anthemis*), with DME of the linear triyne type [150].

## References

[1] Bohlmann F., Burkhardt T., Zdero C. (1973). Naturally Occurring Acetylenes.

[2] Sörensen N.A., Heywood V.H., Harborne J.B., Turner B.L. (1977). Polyacetylenes and conservatism of chemical characters in the Compositae. The Biology and Chemistry of the Compositae.

[3] Bohlmann F., Lam L., Breteler H., Arnason T., Hansen L. (1988). Naturally occurring acetylenes. Chemistry and Biology of Naturally-Occurring Acetylenes and Related Compounds (NOARC).

[4] Minto R.E., Blacklock B.J. (2008). Biosynthesis and function of polyacetylenes and allied natural products. Prog. Lipid Res..

[5] Konovalov D.A. (2014). Medicinal plants. Polyacetylene compounds of plants of the Asteraceae family (Review). Pharm. Chem. J..

[6] Negri R. (2015). Polyacetylenes from terrestrial plants and fungi: Recent phytochemical and biological advances. Fitoterapia.

[7] Greger H., Heywood V.H., Harborne J.B., Turner B.L. (1977). Anthemideae-chemical review. The Biology and Chemistry of the Compositae.

[8] Christensen L.P. (1992). Acetylenes and related compounds in Anthemideae. Phytochemistry.

[9] Greger H., Margaris N., Koedam A., Vokou D. (1982). New chemical markers within *Artemisia* (Compositae-Anthemideae). Aromatic Plants: Basic and Applied Aspects.

[10] Greger H. (1979). Polyacetylene und Sesamine als chemische Merkmale in der *Artemisia absinthium*-Gruppe. Planta Med..

[11] Greger H. (1979). Aromatic acetylenes and dehydrofalcarinone derivatives within the *Artemisia dracunculus* group. Phytochemistry.

[12] Wallnöfer B., Hofer O., Greger H. (1989). Polyacetylenes from the *Artemisia* ‘Vulgares’ group. Phytochemistry.

[13] Wallnöfer B. (1994). Die Polyacetylene in der Artemisia-“Vulgares”-Gruppe (Anthemideae-Compositae).

[14] Bohlmann F., Jente R., Lucas W., Laser J., Schulz H. (1967). Die Biogenese von Polyinen des Tribus Anthemideae. Chem. Ber..

[15] Bu’Lock J.D., Smith G.N. (1967). The origin of naturally-occurring acetylenes. J. Chem. Soc. C.

[16] Bohlmann F., Arndt C., Bornowski H., Kleine K.M. (1961). Über Polyine aus der Familie der Umbelliferen. Chem. Ber..

[17] Hansen L., Boll P.M. (1986). Polyacetylenes in Araliaceae: Their chemistry, biosynthesis and biological significance. Phytochemistry.

[18] Christensen L.P., Brandt K. (2006). Bioactive polyacetylenes in food plants of the Apiaceae family: Occurrence, bioactivity and analysis. J. Pharm. Biomed. Anal..

[19] Christensen L.P. (2011). Aliphatic C_17_- polyacetylenes of the flacarinol type as potential health promoting compounds in food plants of the Apiaceae family. Recent Pat. Food Nutr. Agric..

[20] Chen Y., Peng S., Luo Q., Zhang J., Guo Q., Zhang Y., Chai X. (2015). Chemical and pharmacological progress on polyacetylenes isolated from the family Apiaceae. Chem. Biodiv..

[21] Anet E.F.L.J., Lythgoe B., Silk M.H., Trippett S. (1953). Oenanthotoxin and cicutoxin. Isolation and structures. J. Chem. Soc..

[22] Imai K. (1956). Studies on the essential oil of *Artemisia capillaris* Thunb. III. Antifungal activity of the essential oil (3). Structure of antifungal principle, capillin. J. Pharm. Soc. Jap. (Yakugaku Zasshi).

[23] Cascon S.C., Mors W.B., Tursch B.M., Aplin R.T., Durham L.J. (1965). Ichthyothereol and its acetate, the active polyacetylene constituents of *Ichthyothere terminalis* (Spreng.) Malm: A fish poison from the lower Amazon. J. Am. Chem. Soc.

[24] Xie Q., Wang C. (2022). Polyacetylenes in herbal medicine: A comprehensive review of its occurrence, pharmacology, toxicology, and pharmacokinetics (2014–2021). Phytochemistry.

[25] Yano K. (1983). Insect antifeeding phenylacetylenes from growing buds of *Artemisia capillaris*. J. Agric. Food Chem..

[26] Arnason J.T., Bourque G.J., Madhosingh C., Orr W. (1986). Disruption of membrane function in *Fusarium culmorum* by an acetylenic allelochemical. Biochem. Syst. Ecol..

[27] Engelmeier D., Hadacek F., Hofer O., Lutz-Kutschera G., Nagl M., Wurz G., Greger H. (2004). Antifungal 3-butylisocoumarins from Asteraceae-Anthemideae. J. Nat. Prod..

[28] Whelan L.C., Ryan M.F. (2004). Effects of the polyacetylene capillin on human tumor cell lines. Anticancer Res..

[29] Nakamura Y., Ohto Y., Murakami A., Jiwajinda S., Ohigashi H. (1998). Isolation and identification of acetylenic spiroketal enol ethers from *Artemisia lactiflora* as inhibitors of superoxide generation induced by a tumor promoter in differentiated HL-60 cells. J. Agric. Food Chem..

[30] Nakamura Y., Kawamoto N., Ohto Y., Torikai K., Murakami A., Ohigashi H. (1999). A diacetylenic spiroketal enol ether epoxid, AL-1, from *Artemisia lactiflora* inhibits 12-*O*-tetradecanoylphorbol-13-acetate-induced tumor promotion possibly by suppression of oxidative stress. Cancer Lett..

[31] Chen L., Xu H.H., Wu Y.L. (2005). Synthesis of spiroketal enol ethers related to tonghaosu and their insecticidal activities. Pest Manag. Sci..

[32] Calzado M.A., Lüdi K.S., Fiebich B., Ben-Neriah Y., Bacher S., Munoz E., Ballero M., Prosperini S., Appendino G., Schmitz M.L. (2005). Inhibition of NF-κB activation and expression of inflammatory mediators by polyacetylene spiroketals from *Plagius flosculosus*. Biochim. Biophys. Acta.

[33] Casu L., Bonsignore L., Pinna M., Casu M., Floris C., Gertsch J., Cottiglia F. (2006). Cytotoxic diacetylenic spiroketal enol ethers from *Plagius flosculosus*. J. Nat. Prod..

[34] Rahman M.A.A., Cho S.C., Song J., Mun H.T., Moon S.S. (2007). Dendrazawaynes A and B, antifungal polyacetylenes from *Dendranthema zawadskii* (Asteraceae). Planta Mrd..

[35] Haouras D., Guido F., Monia B.H.K., Habib B.H.M. (2011). Identification of an insecticidal polyacetylene derivative from *Chrysanthemum macrotum* leaves. Ind. Crop Prod..

[36] Álvarez Á.L., Habtemariam S., Abdel Moneim A.E., Melón S., Dalton K.P., Parra F. (2015). A spiroketal-enol ether derivative from *Tanacetum vulgare* selectively inhibits HSV-1 and HSV-2 glycoprotein accumulation in vero cells. Antivir. Res..

[37] Kemp M.S. (1978). Falcarindiol: An antifungal polyacetylene from *Aegopodium podagraria*. Phytochemistry.

[38] Garrod B., Lewis B.G. (1979). Location of the antifungal compound falcarindiol in carrot root tissue. Trans. Br. Mycol. Soc..

[39] Garrod B., Lea E.J.A., Lewis B.G. (1979). Studies on the mechanism of action of the antifungal compound falcarindiol. New Phytol..

[40] De Wit P.J.G.M., Kodde E. (1981). Induction of polyacetylenic phytoalexins in *Lycopersicon esculentum* after inoculation with *Cladosporium fulvum* (syn. *Fulvia fulva*). Physiol. Plant Pathol..

[41] Muir A.D., Cole A.L.J., Walker J.R.L. (1982). Antibiotic compounds from New Zealand plants. I. Falcarindiol, an anti-dermatophyte agent from *Schefflera digitata*. Planta Med..

[42] Wang Y., Toyota M., Krause F., Hamburger M., Hostettmann K. (1990). Polyacetylenes from *Artemisia borealis* and their biological activities. Phytochemistry.

[43] Wang Y., Toyota M., Krause F., Hamburger M., Hostettmann K. (1990). Antifungal and larvicidal polyacetylenes from *Artemisia borealis*. Planta Med..

[44] Matsuura H., Saxena G., Farmer S.W., Hancock R.E.W., Towers G.H.N. (1996). Antibacterial and antifungal polyine compounds from *Glehnia littoralis* ssp. leiocarpa. Planta Med..

[45] Yoon M.Y., Choi G.J., Choi Y.H., Jang K.S., Cha B., Kim J.C. (2011). Antifungal activity of polyacetylenes isolated from *Cirsium japonicum* roots against various phytopathogenic fungi. Ind. Crop Prod..

[46] Wu H.B., Guo P.X., Ma L.H., Li X.M., Liu T.T. (2021). Nematicidal, antifungal and insecticidal activities of *Artemisia halodendron* extrats. Ind. Crops Prod..

[47] Guillet G., Philogéne B.J.R., O’Meara J., Durst T., Arnason J.T. (1997). Multiple modes of insecticidal action of three classes of polyacetylene derivatives from *Rudbeckia hirta*. Phytochemistry.

[48] Eckenbach U., Lampman R.L., Seigler D.S., Ebinger J., Novak K.J. (1999). Mosquitocidal activity of acetylenic compounds from *Cryptotaenia canadensis*. J. Chem. Ecol..

[49] Uhlenbroek J.H., Bijloo J.D. (1958). Investigations on nematicides: I. Isolation and structure of a nematicidal principle occurring in *Tagetes* roots. Rec. Trav. Chim. Pays-Bas.

[50] Gommers F.J., Bakker J., Lam L., Breteler H., Arnason T., Hansen L. (1988). Mode of action of _-terthienyl and related compounds may explain the suppressant effects of *Tagetes* species on populations of free living endoparasitic plant nematodes. Chemistry and Biology of Naturally-Occurring Acetylenes and Related Compounds (NOARC).

[51] Liu G., Lai D., Liu Q.Z., Zhou L., Liu Z.L. (2016). Identification of nematicidal constituents of *Notopterygium incisum* rhizomes against *Bursaphelenchus xylophilus* and *Meloidogyne incognita*. Molecules.

[52] Matsunaga H., Katano M., Yamamoto H., Fujito H., Mori M., Takata K. (1990). Cytotoxic activity of polyacetylene compounds in *Panax ginseng* C. A. Meyer. Chem. Pharm. Bull..

[53] Lechner D., Stavri M., Oluwatuyi M., Pereda-Miranda R., Gibbons S. (2004). The anti-staphylococcal activity of *Angelica dahurica* (Bai Zhi). Phytochemistry.

[54] Hinds L., Kenny O., Hossain M.B., Walsh D., Sheehy E., Evans P., Gaffney M., Rai D.K. (2017). Evaluating the antibacterial properties of polyacetylene and glucosnolate compounds with further identification of their presence within various carrot (*Daucus carota*) and broccoli (*Brassica oleracea*) cultivars using high-performance liquid chromatography with a diode array detector and ultra performance liqhid chromatography-tandem mass spectrometry analyses. J. Agric. Food Chem..

[55] Setzer W.N., Green T.J., Whitaker K.W., Moriarity D.M., Yancey C.A., Lawton R.O., Bates R.B. (1995). A cyctotoxic diacetylene from *Dendropanax arboreus*. Planta Med..

[56] Bernart M.W., Cardellina J.H., Balaschak M.S., Alexander M.R., Shoemaker R.H., Boyd M.R. (1996). Cytotoxic falcarinol oxylipins from *Dendropanax arboreus*. J. Nat. Prod..

[57] Stavri M., Ford C.H.J., Bucar F., Streit B., Hall M.L., Williamson R.T., Mathew K.T., Gibbons S. (2005). Bioactive constituents of *Artemisia monosperma*. Phytochemistry.

[58] Zidorn C., Jöhrer K., Ganzera M., Schubert B., Sigmund E.M., Mader J., Greil R., Ellmerer E.P., Stuppner H. (2005). Polyacetylenes from the Apiaceae vegetables carrot, celery, fennel, parsley, parsnip and their cytotoxic activities. J. Agric. Food Chem..

[59] Dembitsky V.M. (2006). Anticancer activity of natural and synthetic acetylenic lipids. Lipids.

[60] Purup S., Larsen E., Christensen L.P. (2009). Differential effects of falcarinol and related aliphatic C_17_-polyacetylenes on intestinal cell proliferation. J. Agric. Food Chem..

[61] Kuklev D.V., Domb A.J., Dembitsky V.M. (2013). Bioactive acetylenic metabolites. Phytomedicine.

[62] Jin H.R., Zhao J., Zhang Z., Liao Y., Wang C.Z., Huang W.H., Li S.P., He T.C., Yuan C.S., Du W. (2012). The antitumor natural compound falcarindiol promotes cancer cell death by inducing endoplasmic reticulum stress. Cell Death Dis..

[63] Jin H.R., Liao Y., Li X., Zhang Z., Zhao J., Wang C.Z., Huang W.H., Li S.P., Yuan C.S., Du W. (2014). Anticancer compound oplopantriol A kills cancer cells through inducing ER stress and BH3 proteins Bim and Noxa. Cell Death Dis..

[64] Thomas C.A., Allen E.H. (1970). An antifungal polyacetylene compound from *Phytophthora*-infected safflower. Phytopathology.

[65] Hargreaves J.A., Mansfield J.W., Coxon D.T., Price K.R. (1976). Wyerone epoxide as a phytoalexin in *Vicia faba* and its metabolism by *Botrytis cinerea* and *B. fabae* in vitro. Phytochemistry.

[66] Gommers F.J. (1972). Increase of the nematicidal activity of A-terthienyl and related compounds by light. Nematologica.

[67] Camm E.L., Towers G.H.N., Mitchell J.C. (1975). UV-mediated antibiotic activity of some Compositae species. Phytochemistry.

[68] Binns S.E., Purgina B., Bergeron C., Smith M.L., Ball L., Baum B.R., Arnason J.T. (2000). Light-mediated antifungal activity of *Echinacea* extracts. Planta Med.

[69] Ichihara K.I., Kawai T., Noda M. (1978). Polyacetylenes of *Solidago altissima*. Agric. Biol. Chem..

[70] Kobayashi A., Morimoto S., Shibata Y., Yamashita K., Numata M. (1980). C_10_-Polyacetylenes as allelopathic substances in dominants in early stages of secondary succession. J. Chem. Ecol..

[71] Yano K., Ishizu T. (1994). Capillen, a seed germination inhibitor from *Artemisia capillaris* roots. Phytochemistry.

[72] Christensen L.P. (1998). Biological activities of naturally occurring acetylenes and related compounds from higher plants. Recent Res. Dev. Phytochem..

[73] Christensen L.P. (2020). Bioactive C_17_ and C_18_ acetylenic oxylipins from terrestrial plants as potential lead compounds for anticancer drug development. Molecules.

[74] Siddiq A., Dembitsky V. (2008). Acetylenic anticancer agents. Anti-Cancer Agents Med. Chem..

[75] Lai J.X., Dai S.F., Xue B.X., Zhang L.H., Chang Y., Yang W., Wu H.H. (2023). Plant polyacetylenoids: Phytochemical, analytical and pharmacological updates. Arab. J. Chem..

[76] Kelsey R.G., Shafizadeh F. (1979). Sesquiterpene lactones and systematics of the genus *Artemisia*. Phytochemistry.

[77] Marco J.A., Barbera O., Rahman A.u. (1990). Natural poducts from the genus *Artemisia* L. Studies in Natural Products Chemistry.

[78] Tan R.X., Zheng W.F., Tang H.Q. (1998). Biologically active substances from the genus *Artemisia*. Planta Med..

[79] Bora K.S., Sharma A. (2011). The genus *Artemisia*: A comprehensive review. Pharm. Biol..

[80] Martínez M.J.A., DelOlmo L.M.B., Ticona L.A., Benito P.B., Rahman A.u. (2012). The *Artemisia* L. genus: A review of bioactive sesquiterpene lactones. Studies in Natural Products Chemistry.

[81] Abad M.J., Bedoya L.M., Apaza L., Bermejo P. (2012). The *Artemisia* L. genus: A review of bioactive essential oils. Molecules.

[82] Ivanescu B., Miron A., Corciova A. (2015). Sesquiterpene lactones from *Artemisia* genus: Biological activities and methods of analysis. J. Anal. Methods Chem..

[83] Sainz P., Cruz-Estrada Á., Díaz C.E., Gonzáles-Coloma A. (2017). The genus *Artemisia*: Distribution and phytoc hemistry in the Iberian peninsula and the Canary and Balearic islands. Phytochem. Rev..

[84] Bisht D., Kumar D., Kumar D., Dua K., Kumar Chellappan D. (2021). Phytochemistry and pharmacological activity of the genus *Artemisia*. Arch. Pharm. Res..

[85] Stavholt K., Sörensen N.A. (1950). Studies related to naturally-occurring acetylene compounds. V. Dehydro matricaria ester (methyl *n*-decene triynoate) from the essential oil of *Artemisia vulgaris* L. Acta Chem. Scand..

[86] Harada R., Iwasaki M. (1982). Volatile components of *Artemisia capillaris*. Phytochemistry.

[87] Bohlmann F., Burkhardt T. (1969). Über die Biogenese von C_17_-Polyinen. Chem. Ber..

[88] Bohlmann F., Rode K.M. (1968). Notiz über die Polyine aus *Pittosporum buchanani* Hook. fil. Chem. Ber..

[89] Seger C., Godejohann M., Spraul M., Stuppner H., Hadacek F. (2006). Reaction product analysis by high-performance liquid chromatography-solid-phase extraction-nuclear magnetic resonance: Application to the absolute configuration determination of naturally-occurring polyyne alcohols. J. Chromatogr. A.

[90] Lemmich E. (1981). The absolute configuration of the acetylenic compound falcarindiol. Phytochemistry.

[91] Wang Q., Hao J., Gong J., Bao W. (2020). Isolation and structure elucidation of two new compounds from *Artemisia ordosica* Krasch. Nat. Prod. Res..

[92] Jin L., Zhou W., Li R., Jin M., Jin C., Sun J., Li G. (2021). A new polyacetylene and other constituents with anti-inflammatory activity from *Artemisia halodendron*. Nat. Prod. Res..

[93] Zhao Y., Geng C.A., Sun C.L., Ma Y.B., Huang X.Y., Cao T.W., He K., Wang H., Zhang X.M., Chen J.J. (2014). Polyacetylenes and anti-hepatitis B virus active constituents from *Artemisia capillaris*. Fitoterapia.

[94] Geng C.A., Huang X.Y., Chen X.L., Ma Y.B., Rong G.Q., Zhao Y., Zhang X.M., Chen J.J. (2015). Three new anti-HB V active constituents from the traditional Chinese herb Yin-Chen (*Artemisia scoparia*). J. Ethnopharmacol..

[95] Jakupovic J., Tan R.X., Bohlmann F., Jia Z.J., Huneck S. (1991). Acetylenes and other constituents from *Artemisia dracunculus*. Planta Med..

[96] Bohlmann F., Arndt C., Bornowski H., Jastrow H., Kleine K.M. (1962). Neue Polyine aus dem Tribus Anthemideae. Chem. Ber..

[97] Bohlmann F., Ehlers D. (1977). Ein neues *p*-Hydroxyacetophenon-Derivat aus *Artemisia monosperma*. Phytochemistry.

[98] Hu J., Jia Z., Bai S. (1998). Two new polyacetylenes from *Artemisia eriopoda*. Planta Med..

[99] Bohlmann F., Niedballa U., Rode K.M. (1966). Über neue Polyine mit C_17_-Kette. Chem. Ber..

[100] Stavri M., Mathew K.T., Gibson T., Williamson R.T., Gibbons S. (2004). New constituents of *Artemisia monosperma*. J. Nat. Prod..

[101] Löfgren N. (1949). Centaur X and centaur Y. Two unknown substances in *Centaurea*-species. Acta Chem. Scand..

[102] Bohlmann F., Inhoffen E., Herbst P. (1957). Die Konstitution der Polyin-Kohlenwasserstoffe aus *Centaurea cyanus* und *Artemisia vulgaris*. Chem. Ber..

[103] Bohlmann F., Rode K.M. (1967). Die Inhaltsstoffe aus *Artemisia selengensis* auct. Chem. Ber..

[104] Bohlmann F., Mannhardt H.J. (1955). Zur Konstitution des Dehydromatricariaesters aus *Artemisia vulgaris*. Chem. Ber..

[105] Stene-Sörensen J., Bruun T., Holme D., Sörensen N.A. (1954). Studies related to naturally occurring acetylene compounds XIII: The occurrence of *trans*-methyl-*n*-dec-2-en-4:6:8-triyonate in the genus *Tripleurospermum* Schultz Bipontinus. Acta Chem. Scand..

[106] Drake D., Lam J. (1974). Polyacetylenes of *Artemisia vulgaris*. Phytochemistry.

[107] Bohlmann F., von Kap-herr W., Fanghänel L., Arndt C. (1965). Über einige neue Inhaltsstoffe aus dem Tribus Anthemideae. Chem. Ber..

[108] Greger H. (1978). A new acetylenic ester from *Artemisia absinthium*. Phytochemistry.

[109] Liu T.T., Wu H.b., Wu H.b., Zhang J. (2019). Wormwood (*Artemisia absinthium*) as a promising nematicidal and antifungal agent: Chemical composition, comparison of extraction techniques and bioassay-guided isolation. Ind. Crop Prod..

[110] Yamari A., Boriky D., Bouamrani M.L., Blaghen M., Talbi M. (2004). A new thiophen actylene from *Artemisia absithium*. J. Chin. Chem. Soc..

[111] Bohlmann F., Kleine K.M., Arndt C., Köhn S. (1965). Neue Inhaltsstoffe der Gattung *Anthemis*. Chem. Ber..

[112] Bohlmann F., Mannhardt H.J., Viehe H.G. (1955). Synthese des Polyinketons aus *Artemisia vulgaris*. Chem. Ber..

[113] Bohlmann F., Karl W., Zeisberg R. (1970). Über die Biogenese von Acetylenverbindungen aus dem Tribus Anthemideae. Chem. Ber..

[114] Marco J.A., Sanz-Cervera J.F., Sancenón F., Arnó M., Vallés-Xirau J. (1994). Sesquiterpene lactones and acetylenes from *Artemisia reptans*. Phytochemistry.

[115] Bohlmann F., Zdero C. (1972). Constituents of *Artemisia afra*. Phytochemistry.

[116] Bohlmann F., Arndt C., Bornowski H. (1960). Über weitere Polyine aus dem Tribus *Anthemideae* L. Chem. Ber..

[117] Bohlmann F., Kleine K.M. (1964). Über zwei neue Polyinacetate. Chem. Ber..

[118] Bohlmann F., Arndt C., Kleine K.M., Bornowski H. (1965). Die Acetylenverbindungen der Gattung *Echinops* L. Chem. Ber..

[119] Manns D., Hartmann R. (1992). Annuadiepoxide, a new polyacetylene from the aerial parts of *Artemisia annua*. J. Nat. Prod..

[120] Ivarsen E., Fretté X.C., Christensen K.B., Christensen L.P., Engberg R.M., Grevsen K., Kjaer A. (2014). Bioassay-guided chromatographic isolation and identification of antibacterial compounds from *Artemisia annua* L. that inhibit *Clostridium perfringens* growth. J. AOAC Internat..

[121] Jakupovic J., Chau-Thi T.V., Warning U., Bohlmann F., Greger H. (1986). 11β,13-Dihydroguaianolides from *Artemisia douglasiana* and a thiophene acetylene from *A. schmidtiana*. Phytochemistry.

[122] Bohlmann F., Florentz G. (1966). Über die Biogenese der Spiroketalenolätherpolyine. Chem. Ber..

[123] Bohlmann F., Kapteyn H.G. (1966). Über die Polyine aus *Chrysanthemum serotinum* L. Chem. Ber..

[124] Birnecker W., Wallnöfer B., Hofer O., Greger H. (1988). Relative and absolute configurations of two naturally occurring acetylenic spiroketal enol ether epoxides. Tetrahedron.

[125] Ma L., Ge F., Tang C.P., Ke C.Q., Li X.Q., Althammer A., Ye Y. (2011). The absolute configuration determination of naturally occurring diacetylenic spiroacetal enol ethers from *Artemisia lactiflora*. Tetrahedron.

[126] Bohlmann F., Rode K.M. (1966). Über die Inhaltsstoffe von *Artemisia pedemontana*. Chem. Ber..

[127] Bohlmann F., Herbst P., Arndt C., Schönowsky H., Gleinig H. (1961). Über einen neuen Typ von Polyacetylenverbindungen aus verschiedenen Vertretern des Tribus *Anthemideae* L. Chem. Ber..

[128] Bohlmann F., Arndt C., Bornowski H., Kleine K.M., Herbst P. (1964). Neue Acetylenverbindungen aus *Chrysanthemum*-Arten. Chem. Ber..

[129] Bohlmann F., Ates N., Jakupovic J., King R.M., Robinson H. (1982). Types of sesquiterpenes from *Artemisia douglasiana*. Phytochemistry.

[130] Wang K.D.G., Wang J., Xie S.S., Li Z.R., Kong L.Y., Luo J. (2016). New naturally occurring diacetylenic spiroacetal enol ethers from *Artemisia selengensis*. Tetrahedron Lett..

[131] Tan R.X., Jia Z.J., Zhao Y., Feng S.L. (1992). Sesquiterpenes and acetylenes from *Artemisia feddei*. Phytochemistry.

[132] Bohlmann F., Bornowski H., Schönowsky H. (1962). Über heterocyclisch substituierte Acetylenverbindungen aus dem Tribus *Anthemideae* L. Chem. Ber..

[133] Hofer O., Wallnöfer B., Widhalm M., Greger H. (1988). Naturally occurring thienyl-substituted spiroacetalenol ethers from *Artemisia ludoviciana*. Liebigs Ann. Chem..

[134] Xiao M.T., Luo D.W., Ke Z., Ye J., Tu P.F. (2014). A novel polyacetylene from the aerial parts of *Artemisia lactiflora*. Phytochem. Lett..

[135] Harada R. (1956). The structure of capillon. J. Chem. Soc. Jpn..

[136] Harada R. (1957). The structure of capillen. J. Chem. Soc. Jpn..

[137] Miyazawa M., Kameoka H. (1975). A new polyacetylene from *Artemisia capillaris*. Phytochemistry.

[138] Miyazawa M., Kameoka H. (1975). Capillanol: A new acetylenic alcohol from *Artemisia capillaris*. Phytochemistry.

[139] Miyazawa M., Kameoka H. (1976). Neocapillen, a new acetylenic hydrocarbon from *Artemisia* capillaris. Phytochemistry.

[140] Bohlmann F., Kleine K.M. (1962). Die Polyine aus *Chrysanthemum frutescens* und *Artemisia dracuncus* L. Chem. Ber..

[141] Islam M.N., Choi K.J., Jung H.A., Oh S.H., Choi J.S. (2016). Promising anti-diabetic potential of capillin and capillinol isolated from *Artemisia capillaris*. Arch. Pharm. Res..

[142] Bohlmann F., Zdero C. (1971). Über zwei neue Phenylpolyine aus *Anthemis fuscata* Brot. Chem. Ber..

[143] Ulubelen A., Öksuz S. (1984). Capillarin and scoparone from *Artemisia lamprocaulos*. J. Nat. Prod..

[144] Harada R., Noguchi S., Sugiyama N. (1960). Structure of capillarin. J. Chem. Soc. Jpn..

[145] Greger H., Bohlmann F. (1979). 8-Hydroxycapillarin-ein neues Isocumarin aus *Artemisia dracunculus*. Phytochemistry.

[146] Riggins C.W., Clausen T.P. (2003). Root acetylenes from *Artemisia arctica*. Biochem. Syst. Ecol..

[147] Mallabaev A., Yagudaev M.R., Saitbaeva I.M., Sidyakin G.P. (1970). The isocoumarin artemidin from *Artemisia dracunculus*. Chem. Nat. Comp..

[148] Mallabaev A., Saitbaeva I.M., Sidyakin G.P. (1970). Structure of the isocoumarin artemidin. Chem. Nat. Comp..

[149] Mallabaev A., Sidyakin G.P. (1974). Artemidiol—A new isocoumarin from *Artemisia dracunculus*. Chem. Nat. Comp..

[150] Bohlmann F., Zdero C. (1970). Die Inhaltsstoffe aus *Anthemis fuscata* Brot. Chem. Ber..

[151] Greger H., Bohlmann F., Zdero C. (1977). Neue Isocumarine aus *Artemisia dracunculus*. Phytochemistry.

[152] Meepagala K.M., Sturtz G., Wedge D.E. (2002). Antifungal constituents of the essential oil fraction of *Artemisia dracunculus* L. var. dracunculus. J. Agric. Food Chem..

[153] Yano K. (1975). Variation in acetylene content of different ecotypes of *Artemisia capillaris*. Phytochemistry.

[154] Yano K. (1986). Relationships between chemical structure of phenylalkynes and their antifeeding activity for larvae of a cabbage butterfly. Insect Biochem..

[155] Yano K. (1987). Minor components from growing buds of *Artemisia capillaris* that act as insect antifeedants. J. Agric. Food Chem..

[156] Saleh M.A. (1984). An insecticidal diacetylene from *Artemisia monosperma*. Phytochemistry.

[157] Zhang Z., Guo S.S., Zhang W.J., Geng Z.F., Liang J.Y., Du S.S., Wang C.F., Deng Z.W. (2017). Essential oil and polyacetylenes from *Artemisia ordosica* and their bioactivities against *Tribolium castaneum* Herbst (Coleoptera: Tenebrionidae). Ind. Crop Prod..

[158] Yin B.L., Fan J.F., Gao Y., Wu Y.L. (2003). Progress in molecular diversity of tonghaosu and its analogs. Arkivoc.

[159] Barrero A.F., Herrador del Pino M.M., González Portero A., Arteaga Burón P., Arteaga J.F., Alquézar J.B., Díaz E., González-Coloma A. (2013). Terpenes and polyacetylenes from cultivated *Artemisia granatensis* Bois. (Royal chamomile) and their defensive properties. Phytochemistry.

[160] Zhai D.D., Zhong J.J. (2010). Simultaneous analysis of three bioactive compounds in *Artemisia annua* hairy root cultures by reversed-phase high-performance liquid chromatography-diode array detector. Phytochem. Anal..

[161] Kogiso S., Wada K., Munakata K. (1976). Isolation of nematicidal polyacetylenes from *Carthamus tinctorius* L. Agric. Biol. Chem..

[162] Kogiso S., Wada K., Munakata K. (1976). Nematicidal polyacetylenes, 3Z,11E-and 3E,11E-trideca-1,3,11-triene-5,7,9-triyne from *Carthamus tinctorius* L. Tetrahedron Lett..

[163] Kim H., Lee Y.H., Kim S.L. (1988). A possible mechanism of polyacetylene: Membrane cytotoxicity. Korean J. Tooxicol..

[164] Jung H.J., Min B.S., Park J.Y., Kim Y.H., Lee H.K., Bae K.H. (2002). Gymnasterkoreaynes A-F, cytotoxic polyacetylenes from *Gymnaster koraiensis*. J. Nat. Prod..

[165] Makuda Y., Asada K., Satoh R., Takada K., Kitajama J. (2015). Capillin, a major constituent of *Artemisia capillaris* Thunb. flowerr essential oil, induces apoptosis through the mitochondrial pathway in human leukemia HL-60 cells. Phytomedicine.

[166] Avato P., Vitali C., Mongelli P., Tava A. (1997). Antimicrobial activity of polyacetylenes from *Bellis perennis* and their synthetic derivatives. Planta Med..

[167] Metzger B.T., Barnes D.M., Reed J.D. (2008). Purple carrot (*Daucus carota* L.) polyacetylenes decrease lipopolysaccharide-induced expression of inflammatory proteins in macrophage and endothelial cells. J. Agric. Food Chem..

[168] Yamamoto M., Ogawa K., Morita M., Fukuda K., Komatsu Y. (1996). The herbal medicine inchin-ko-to inhibits liver cell apoptosis induced by transforming growth factor *β*1. Hepatology.

[169] Vallés J., Garcia S., Hidalgo O., Martin J., Pellicer J., Sanz M., Garnatje T. (2011). Biology, genome evolution, biotechnological issues and research including applied perspectives in Artemisia (Asteraceae). Adv. Bot. Res..

[170] Ehrendorfer F. (1964). Notizen zur Cytotaxonomie und Evolution der Gattung *Artemisia*. Österr. Bot. Z..

[171] Gutermann W.E. (1979). Systematik und Evolution Einer Alten, Dysploid-Polyploiden Oreophyten-Gruppe: *Artemisia mutellina* und Ihre Verwandten (Asteraceae: Anthemideae). Ph.D. Thesis.

[172] Bohlmann F., Ang W., Trinks C., Jakupovic J., Huneck S. (1985). Dimeric guaianolides from *Artemisia sieversiana*. Phytochemistry.

[173] Martínez V., Barberá O., Sánchez-Parareda J., Marco J.A. (1987). Phenolic acetylenic metabolites from *Artemisia assoana*. Phytochemistry.

[174] Greger H., Hofer O. (1980). New unsymmetrically substituted tetrahydrofurofuran lignans from *Artemisia absinthium*. Assignment of the relative stereochemistry by lanthanide-induced chemical shifts. Tetrahedron.

[175] Greger H. (1981). Sesamin-type lignans as chemical markers within *Artemisia*. Biochem. Syst. Ecol..

[176] Tulake A., Jiang Y., Tu P.F. (2012). Nine lignans from *Artemisia absinthium* L. J. Chin. Pharm. Sci..

[177] Greger H., Hofer O., Nikiforov A. (1982). New sesquiterpene-coumarin ethers from *Achillea* and *Artemisia* species. J. Nat. Prod..

[178] Bohlmann F., Zdero C., Faass U. (1973). Über die Inhaltsstoffe von *Artemisia fragrans* Willd. Chem. Ber..

